# Genetic dissection of the miR-200–Zeb1 axis reveals its importance in tumor differentiation and invasion

**DOI:** 10.1038/s41467-018-07130-z

**Published:** 2018-11-07

**Authors:** Alexandra C. Title, Sue-Jean Hong, Nuno D. Pires, Lynn Hasenöhrl, Svenja Godbersen, Nadine Stokar-Regenscheit, David P. Bartel, Markus Stoffel

**Affiliations:** 1 Institute of Molecular Health Sciences, ETH Zurich, Otto-Stern-Weg 7, 8093 Zürich, Switzerland; 2 Competence Center Personalized Medicine, ETH Zurich, Voltastrasse 24, 8044 Zürich, Switzerland; 3 Howard Hughes Medical Institute and Whitehead Institute for Biomedical Research, Cambridge, MA 02142 USA; 4 Department of Biology, Massachusetts Institute of Technology, Cambridge, MA 02139 USA; 5 Institute of Animal Pathology (COMPATH), Vetsuisse Faculty, University of Bern, 3012 Bern, Switzerland; 6 Medical Faculty, University of Zurich, 8091 Zurich, Switzerland

## Abstract

The epithelial-to-mesenchymal transition (EMT) is an important mechanism for cancer progression and metastasis. Numerous in vitro and tumor-profiling studies point to the miR-200–Zeb1 axis as crucial in regulating this process, yet in vivo studies involving its regulation within a physiological context are lacking. Here, we show that miR-200 ablation in the *Rip-Tag2* insulinoma mouse model induces beta-cell dedifferentiation, initiates an EMT expression program, and promotes tumor invasion. Strikingly, disrupting the miR-200 sites of the endogenous *Zeb1* locus causes a similar phenotype. Reexpressing members of the miR-200 superfamily in vitro reveals that the miR-200c family and not the co-expressed and closely related miR-141 family is responsible for regulation of *Zeb1* and EMT. Our results thus show that disrupting the in vivo regulation of *Zeb1* by miR-200c is sufficient to drive EMT, thus highlighting the importance of this axis in tumor progression and invasion and its potential as a therapeutic target.

## Abstract (web summary)

In vivo studies on the regulation of the epithelial-mesenchymal transition (EMT) within a physiological context are lacking. Here it is shown that miR-200 ablation in the Rip-Tag2 insulinoma mouse model induces beta-cell dedifferentiation, EMT and tumor invasion, and that disruption of Zeb1 regulation by miR-200c is sufficient to drive EMT.

## Introduction

MicroRNAs (miRNAs) have emerged as important mediators of cellular responses to physiological or pathological stress^1–3^. miRNA-mediated regulation of fundamental biological processes, such as proliferation and apoptosis, can link these regulatory RNAs to cancer-relevant pathways^4^. Tumors ubiquitously exhibit dysregulated miRNA expression patterns, providing useful, albeit correlative, information for tumor classification and prognosis^5^. The pathophysiological relevance of dysregulated miRNAs and their targets in tumor specimens is difficult to interpret as their aberrant expression may be confounded by intra-tumoral heterogeneity, hampering the ability to establish causal relationships between miRNA/target levels and cancer phenotypes. This caveat highlights the importance of genetic studies and rigorous functional experiments that directly assess the consequences of miRNA expression manipulation.

The epithelial-to-mesenchymal transition (EMT) is a complex biological process in which biochemical changes enable epithelial cells to adopt a mesenchymal phenotype, resulting in a loss of cell polarity and increased migratory capacity^6–8^. This program operates in embryonic development, wound healing, tissue regeneration and fibrosis, and all stages of cancer progression, as well as resistance to cytotoxic therapy^9,10^. The EMT program has been well studied in carcinomas, in which it involves distinct molecular processes including the induction of EMT transcription factors (EMT-TFs), changes in expression patterns of cell-surface proteins, cytoskeletal reorganization, degradation of extracellular matrix, and altered expression of specific miRNAs^11–13^. Among the most extensively studied EMT-TFs are those of the ZEB, SNAIL and TWIST families, which have pleiotropic functions that include cell invasion and dissemination, but also influence cell fate, cancer-stem-cell plasticity, oncogenic transformation, therapy resistance, immune evasion, and tumor micro-environment^14–16^. Furthermore, extra- and intracellular signaling (i.e. TGF-β, ERK, MAPK, and Smad) regulates the activity of EMT-TFs^13^. An additional layer of EMT-TF regulation involves miRNA networks, which influence EMT via complex feedback loops with EMT-TFs and establish functional links with other signaling pathways^17,18^.

**Fig. 1:**
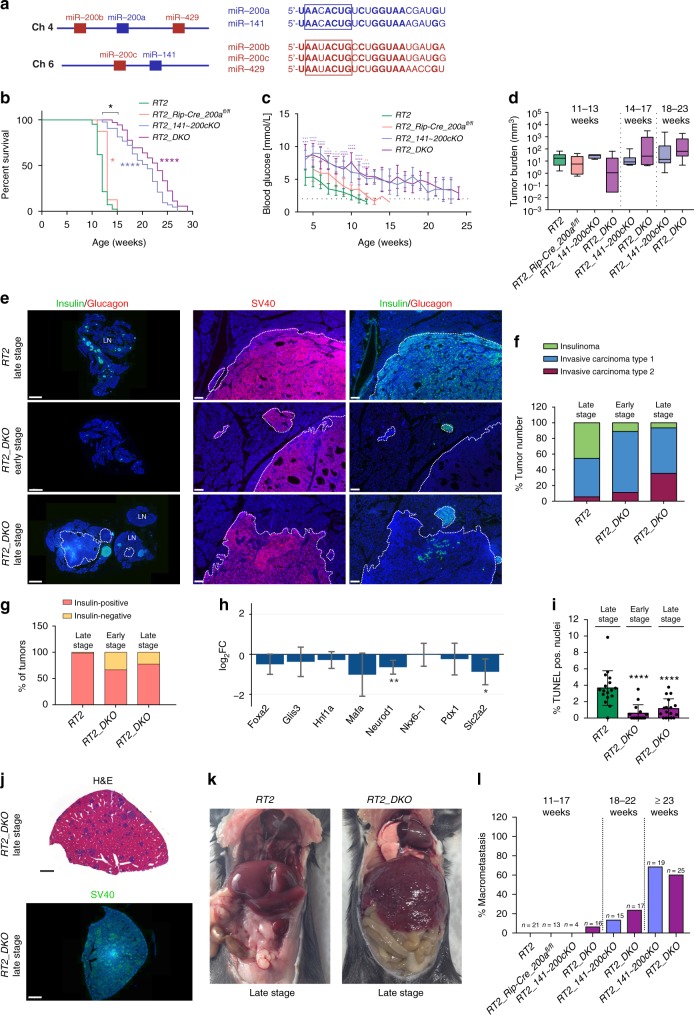
miR-200 ablation promotes tumor growth, malignancy, and invasion. **a** Diagram of murine miR-200 superfamily (seed regions boxed). **b** Percent survival of *RT2* mice with one or both miR-200 genomic clusters ablated (*RT2*, *RT2_Rip-Cre_200a*^*fl/fl*^, *RT2_141~200cKO*, *RT2_DKO*, *n* = 42, 8, 43, 36, respectively). **c** Random-fed blood glucose (*RT2*, *RT2_Rip-Cre_200a*^*fl/fl*^, *RT2_141~200cKO*, *RT2_DKO*, *n* = 11, 7, 11, 13, respectively). Dotted line represents onset of severe hypoglycemia. **d** Tumor burden in different age classes (box, 25th and 75th percentiles; central line, median; left to right: *n* = 9, 5, 4, 6, 9, 7, 11, 14 mice). **e** Representative insulin/glucagon and SV40 immunofluorescence staining of whole pancreas (left; insulin-negative tumors outlined; scale bar = 2 mm) and zoomed-in regions (right; all tumors outlined; scale bar = 100 μm). LN lymph node. **f** Percent tumors per grade (*n* = 5 mice per group; *RT2*, early-stage *RT2_DKO*, late-stage *RT2_DKO*, *n* = 55, 9, 31 tumors respectively). **g** Percent insulin-positive and -negative tumors (*n* = 5 mice per group; *RT2*, early-stage *RT2_DKO*, late-stage *RT2_DKO*, *n* = 67, 6, 31 tumors, respectively). **h** Mean log_2_FC (with 95% confidence intervals) of beta-cell-identity genes in *RT2_DKO* vs. *RT2* islets of 6-week-old mice. **i** Percent of TUNEL-positive nuclei (*n* = 2 mice per group; *RT2*, early-stage *RT2_DKO*, late-stage *RT2_DKO,* *n* = 17, 15, 17 lesions per group). **j** Representative H&E staining and SV40 immunofluorescence staining of late-stage *RT2_DKO* liver (scale bar = 2 mm). **k** Representative images of late-stage *RT2* and *RT2_DKO* mice. **l** Percent mice with macrometastasis in difference age classes. Data in **c**, **i** are plotted as mean ± SD. Significance was evaluated by **b** Mantel Cox test, **c** two-tailed *t* test with Holm−Sidak correction (vs. *RT2*), **d**, **i** one-way ANOVA with Dunnett’s multiple comparisons test (vs. *RT2*) and **h** empirical Bayes method. *P ≤ 0.05; **P ≤ 0.01; ***P ≤ 0.001; ****P ≤ 0.0001

The miR-200 superfamily of miRNAs (referred to as miR-200) is reported to play a central role in EMT of many epithelial cancers^18–21^. Importantly, aberrant expression of miR-200 has been associated with initiation and progression of malignant transformation and metastasis formation^20^. The miR-200 superfamily has five members that derive from two chromosomal locations: miR-200b, -200a and -429 from mouse chromosome 4 (human chromosome 1p36.33) and miR-200c and -141 from chromosome 6 (human chromosome 12p13.31). Based on their seed sequence harboring a single-base difference, members of miR-200 are assigned to two functional families, miR-200a, -141 and miR-200b, -200c, -429 (Fig. 1a)^22^. The activity of these miRNA families (here referred to as miR-141 and miR-200c, respectively) has been linked to the regulation of the EMT-TF Zeb1 (Tcf8, δEf1), which harbors nine conserved miR-200 sites in its 3′UTR. Forced overexpression of miR-200 represses *Zeb1* expression, supporting the prediction of *Zeb1* as a miR-200 target gene^23^. In addition, Zeb1 can bind and transcriptionally repress the promoters of the two miR-200 transcription units, thereby constituting a double-negative feedback loop that has been demonstrated in vitro^20,24,25^.

Although clinical studies show intriguing links between the miR-200–Zeb1 axis, mesenchymal marker expression, and prognostic and therapeutic outcomes^18,20,26–28^, these studies provide correlative results that cannot speak to causality or the roles of the different allelic and functional miRNA subgroups, or to the functional role of *Zeb1* regulation specifically, as opposed to regulation of other miR-200 targets. In this study we therefore take a genetic approach to study the impact of allelic variation of the miR-200 family and its target Zeb1 by selectively inactivating the miR-200 alleles or mutating the miR-200 sites in the endogenous *Zeb1* 3′UTR. Our studies reveal a dose-dependence of miR-200c and exquisite sensitivity of Zeb1 regulation that critically impacts tumor differentiation and invasion phenotypes of a well-established tumor model of pancreatic-islet carcinoma.

## Results

### miR-200 ablation increases tumor progression in *RT2* mice

Our previous findings demonstrating that miR-200 is a potent regulator of beta-cell apoptosis^29^ motivated us to investigate its role in neuroendocrine cancer utilizing the *Rip-Tag2* (*RT2*) insulinoma mouse model^30^. In this model, beta-cell-specific expression of the viral oncogene SV40 T-antigen leads to insulinoma formation through inactivation of the p53 and retinoblastoma (Rb) tumor-suppressor pathways. These tumors progress through a dependable course characterized by the development of insulin-secreting beta-cell tumors, leading to a gradual decrease in blood glucose until symptomatic hypoglycemic levels are reached at ~12 to 14 weeks of age^30–34^. Double knockout of both *mir-200* genomic loci in *RT2* beta-cells (*RT2_DKO*) led to markedly increased survival and elevated blood-glucose levels relative to *RT2* mice (Fig. 1b, c), despite increasing tumor burden (Fig. 1d). Interestingly, loss of the *mir-141~200c* locus contributed more to this phenotype than the *mir-200a~200b~429* locus, as *mir-141~200c*-deficient *RT2* mice (*RT2_141~200cKO*) phenocopied *RT2_DKO* mice, whereas mice lacking only *miR-200a~200b~429* (*RT2_Rip-cre_200a*^*flox/flox*^) resembled *RT2* controls (Fig. 1b–d).

We next sought to investigate morphological and functional changes induced in *mir-200*-deficient mice by comparing tumors collected from *RT2* and *RT2_DKO* mice at an early and a late time point based on the average survival times of the different genotypes. The “late stage” timepoint for RT2 mice was chosen as 11 weeks, just prior to reaching fatal hypoglycemic levels, and the “late stage” timepoint for *RT2_DKO* mice was chosen as 23 weeks as this preceded the age at which declining health and/or hypoglycemia necessitated euthanization (Fig. 1b). An “early stage” of 11 weeks was also selected for *RT2_DKO* mice as an additional timepoint for age-matched comparisons to *RT2*.

At 11 weeks of age, *RT2_DKO* mice had a greater proportion of islets than tumors compared to *RT2* controls (Fig. 1d, e), suggesting a delay in islet hyperplasia and proliferation, which was reflected by decreased EdU incorporation (Supplementary Figure 1a) and lower Ki67, Mcm-2, and PCNA levels in islets of 6-week-old animals (Supplementary Figure 1b). However, using a *RT2* tumor classification system^35^, we determined that the existing tumors were more invasive in *RT2_DKO* than *RT2* mice, with a greater proportion of invasive type 1 and 2 carcinomas relative to noninvasive insulinomas (Fig. 1f). Furthermore, 30% of the tumors of early-stage *RT2_DKO* animals were insulin-negative (vs. 0% in *RT2* mice) (Fig. 1e, g), indicative of beta-cell dedifferentiation and corroborated by the consistent downregulation of beta-cell-identity genes already at 6 weeks of age (Fig. 1h). Apoptosis was also decreased (Fig. 1i), as previously observed in a noncancer setting upon *mir-200* knockout^29^. The decreased proliferation and beta-cell dedifferentiation likely explains why *RT2_DKO* mice had higher blood glucose than *RT2* mice starting already at 4 weeks of age (Fig. 1c). Notably, decreased proliferation, dedifferentiation, and increased resistance to apoptosis are all hallmarks of EMT^7,20,36^, suggesting that the EMT program was already initiated at an early stage. At the late-stage timepoint of 23 weeks *RT2_DKO* mice had more invasive tumors compared to late-stage *RT2* mice as well as to early-stage *RT2_DKO* mice (Fig. 1f). Tumors of late-stage *RT2_DKO* mice were also often insulin-negative (Fig. 1g), providing an explanation for longer survival despite increased tumor burden (Fig. 1b–d). Furthermore, they were more resistant to apoptosis and less proliferative relative to *RT2* late-stage tumors (Fig. 1i; Supplementary Figure 1a), thus retaining characteristics of EMT that had been initiated early on. Finally, late-stage *RT2_DKO* tumors had more instances of vascular invasion (Supplementary Figure 1c, d) than late-stage *RT2* tumors.

In addition, by end-stage both *RT2_141~200cKO* and *RT2_DKO* mice had macrometastases in the liver, which we observed at high penetrance in both *RT2_141~200cKO* and *RT2_DKO* mice, but never in *RT2* controls (Fig. 1j–l). Interestingly, these metastatic lesions were SV40-positive but insulin-negative (Supplementary Figure 1e), suggesting that they might have derived from the poorly differentiated, insulin-negative tumors specifically identified in *RT2_DKO* mice. These results show that genetic ablation of both miR-200 clusters in beta-cells leads to an EMT phenotype, including decreased apoptosis, beta-cell dedifferentiation, increased local and vascular invasion, and tumor progression.

### miR-200 site mutation in Zeb1 phenocopies mir-200 ablation

**Fig. 2:**
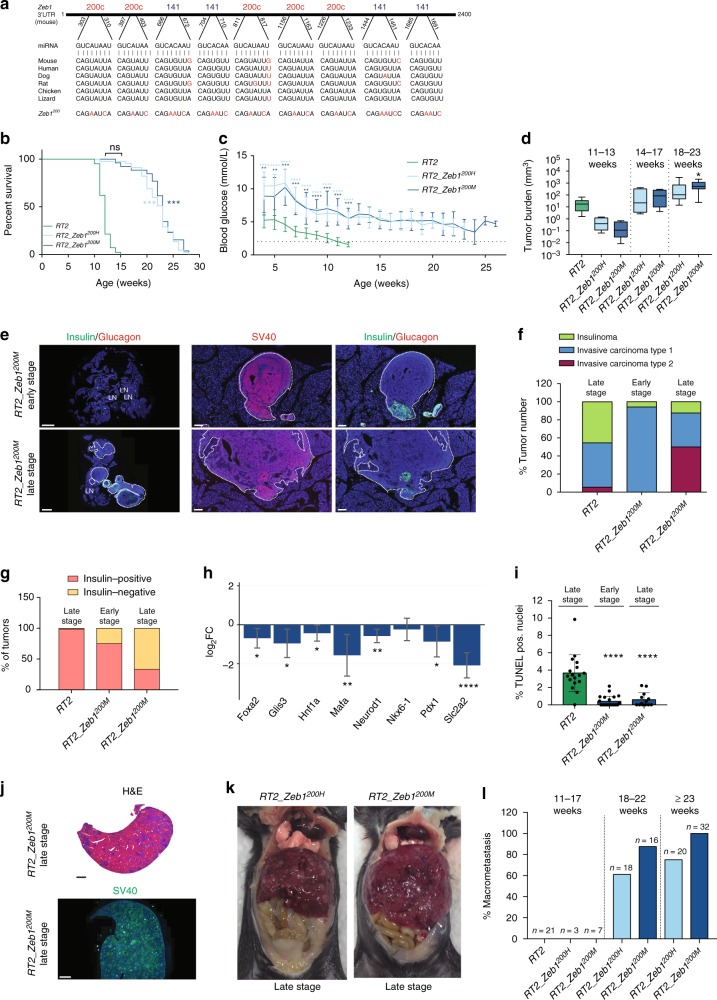
Mutation of miR-200 sites in *Zeb1* is sufficient to phenocopy miR-200 ablation. **a** Murine *Zeb1* 3′UTR, miR-200 site conservation, and mutated miR-200 site sequences. **b** Percent survival of *RT2* mice with *Zeb1* mutation (*RT2*, *RT2_Zeb1*^*200H*^, *RT2_Zeb1*^*200M*^, *n* = 42, 45, 26, respectively). **c** Random-fed blood glucose (*RT2*, *RT2_Zeb1*^*200H*^, *RT2_Zeb1*^*200M*^, *n* = 11, 11, 10, respectively). Dotted line represents onset of severe hypoglycemia. **d** Tumor burden in different age classes (box, 25th and 75th percentiles; central line, median; from left to right: *n* = 9, 4, 4, 4, 5, 11, 12 mice). **e** Representative insulin/glucagon and SV40 immunofluorescence staining of whole pancreas (left; insulin-negative tumors outlined; scale bar = 2 mm) and zoomed-in regions (right; all tumors outlined; scale bar = 100 μm). LN lymph node. **f** Percent tumors per grade (*n* = 5 mice per group; *RT2*, early-stage *RT2_Zeb1*^*200M*^, late-stage *RT2_Zeb1*^*200M*^, *n* = 55, 17, 16 tumors, respectively). **g** Percent insulin-positive and -negative tumors (*n* = 5 mice per group; *RT2*, early-stage *RT2_Zeb1*^*200M*^, late-stage *RT2_Zeb1*^*200M*^, *n* = 67, 12, 15 tumors, respectively). **h** Mean log_2_FC (with 95% confidence intervals) of beta-cell-identity genes in *RT2_Zeb1*^*200M*^ vs. *RT2* islets of 6-week-old mice. **i** Percent TUNEL-positive nuclei (*n* = 2 mice per group; *RT2*, early-stage *RT2_Zeb1*^*200M*^, late-stage *RT2_Zeb1*^*200M*^, *n* = 17, 21, 13 lesions, respectively). **j** Representative H&E and SV40 immunofluorescence staining of late-stage *RT2_Zeb1*^*200M*^ liver (scale bar = 2 mm). **k** Representative images of late-stage *RT2_Zeb1*^*200H*^ and *RT2_Zeb1*^*200M*^ mice. **l** Percent of mice with macrometastasis in difference age classes. **c**, **i** Data are plotted as mean ± SD. Significance was evaluated by **b** Mantel Cox test, **c** two-tailed *t* test with Holm−Sidak correction (vs. *RT2*), **d**, **i** one-way ANOVA with Dunnett’s multiple comparisons (vs. *RT2*) and **h** empirical Bayes method. *P ≤ 0.05; **P ≤ 0.01; ***P ≤ 0.001; ****P ≤ 0.0001

The prevailing model of miRNA function is that they act by repressing the expression of hundreds of targets, whose additive effect can impart strong phenotypic consequences^1,3^. Nonetheless, in some cases, deregulation of just a few targets can explain much of the phenotypic effect observed in genetic models with loss of miRNA function. This possibility is worthy of consideration for Zeb1, which harbors nine conserved miR-200 sites in its 3′UTR (Fig. 2a). To explore the contribution of *Zeb1* deregulation vs. that of hundreds of other mRNAs with conserved miR-200 sites^37^, we generated a knock-in mouse model in which each of the miR-200 sites in the endogenous *Zeb1* locus were mutated (*Zeb1*^*200*^) to disengage *Zeb1* from miR-200 repression (Fig. 2a; Supplementary Figure 2a, b). To study the contribution of Zeb1 to the *RT2_DKO* phenotype, we crossed *Zeb1*^*200*^ into the *RT2* background to generate heterozygous or homozygous mice (*RT2_Zeb1*^*200H*^ *or RT2_Zeb1*^*200M*^, respectively).

Interestingly, *RT2_Zeb1*^*200M*^ mice had an increase in survival resembling that of *RT2_DKO* mice (Fig. 2b) and exhibited a comparable reduction in longitudinal blood-glucose levels to *RT2_DKO* mice (Fig. 2c), along with increased tumor size (Fig. 2d). As observed in *RT2_DKO* animals, early-stage *RT2_Zeb1*^*200M*^ mice had fewer but more invasive tumors compared to *RT2* control mice (Figs. 1e, 2d–f). In addition, early-stage *RT2_Zeb1*^*200M*^ lesions had characteristics indicative of EMT, including dedifferentiation (Fig. 2e, g, h), resistance to apoptosis (Fig. 2i), and decreased proliferation (Supplementary Figure 2c, d). Late-stage *RT2_Zeb1*^*200M*^ mice had a greater proportion of invasive, insulin-negative tumors than late-stage *RT2* or early-stage *RT2_Zeb1*^*200M*^ mice (Fig. 2g). Finally, although tumor burden was similar between *RT2_DKO* and *RT2_Zeb1*^*200M*^ mice (Supplementary Figure 2g), late-stage *RT2_Zeb1*^*200M*^ tumors metastasized to several distal tissues, including liver, lymph nodes, and intestine at a penetrance of almost 100%, even higher than in *RT2_DKO* mice (Figs. 1l, 2j–l). Similar to *RT2_DKO* mice, metastases were also insulin-negative (Supplementary Figure 2h), suggesting migration of dedifferentiated tumor cells. Together, these data revealed that mutating the miR-200 sites of a single target gene phenocopied the ablation of both miR-200 clusters in pancreatic beta-cells, indicating that *Zeb1* is the primary mediator of the dedifferentiation and invasion phenotype. We also investigated the impact of gene dosage of Zeb1: strikingly, mice that had only one mutated *Zeb1* allele (*RT2_Zeb1*^*200H*^) phenotypically resembled that of *RT2_Zeb1*^*200M*^ and *RT2_DKO* mice (Fig. 2b–d, k, l), highlighting the sensitivity of this pathway.

To investigate the broader relevance of the miR-200–Zeb1 axis in epithelial cancers, we crossed the mutated Zeb1 allele into the pancreatic ductal adenocarcinoma (PDAC) “KPC” model^38^. This mouse model harbors mutations in *Kras* and *Trp53* that are frequently mutated in human PDAC and is an extensively used preclinical model of this aggressive cancer^39^. While survival was unaffected in *KPC Zeb1*^*200H*^ and *KPC Zeb1*^*200M*^ mice (Supplementary Figure 2i), they had significantly greater end-stage tumor burden compared to *KPC* mice (Supplementary Figure 2j). All mice had tumors that had progressed to the invasive PDAC stage. Interestingly, however, *KPC Zeb1*^*200H*^ and *KPC Zeb1*^*200M*^ tumors were less differentiated than *KPC* tumors: some were classified as “poorly differentiated” as they had solid regions with complete absence of ducts, but the “moderately differentiated” tumors also had less defined ducts compared to *KPC* tumors (Supplementary Figure 2k, l). Upregulation of *Zeb1* thus led to increased dedifferentiation as observed in the *RT2* background. Furthermore, *KPC Zeb1*^*200M*^ mice had increased liver micrometastasis, reflected by a greater proportion of CK19-positive cells compared to *KPC* mice (Supplementary Figure 2m, n). Collectively, these data emphasize the importance of the miR-200–Zeb1 axis in the progression of other epithelial cancers as well.

### mir-200KO or Zeb1^200^ mutation perturbs target network

**Fig. 3:**
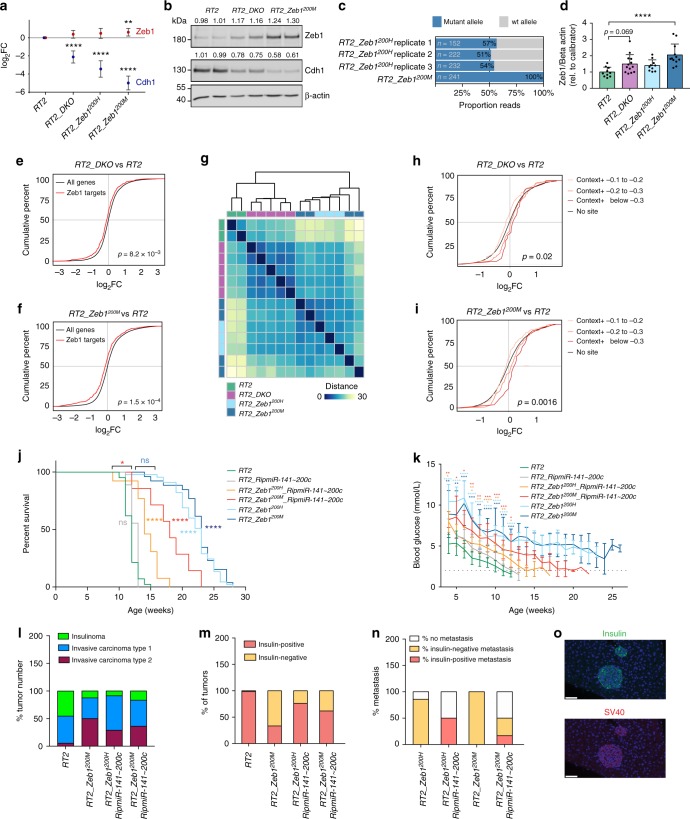
Effect of *miR-200KO* and *Zeb1*^*200*^ mutations on Zeb1 and downstream target regulation. **a** Mean log_2_FC (with 95% confidence intervals) of *Cdh1* and *Zeb1* in islets. **b** Immunoblots evaluating ZEB1 and CDH1 in islets (*n* = 2 mice per lane). Quantification is normalized to β-actin and relative to *RT2*. **c** Allelic expression of mutant *Zeb1* allele in three *RT2_Zeb1*^*200H*^ mice. **d** ZEB1 quantification in end-stage tumors (immunoblots in Supplementary Figure 3b) (*n* = 10−13 tumors), normalized to β-actin and calibrator. **e**, **f** Cumulative distributions of log_2_FC of Zeb1 targets in islets of **e** *RT2_DKO* vs. *RT2* and **f** *RT2_Zeb1*^*200M*^ vs. *RT2*. **g** Hierarchical clustering and heat-map analysis of Zeb1 targets in islets (scale bar represents Euclidian distance between samples). **h**, **i** Cumulative distributions of log_2_FC of miR-200c predicted targets subdivided by context+ score in islets of **h** *RT2_DKO* vs. *RT2* and **i** *RT2_Zeb1*^*200M*^ vs. *RT2* mice. *P* values shown are for context+ <–0.3. **j** Percent survival of *RT2* mice with WT or mutant Zeb1 3′UTR and endogenous or overexpressed miR-200 levels (*RT2*, *RT2_RipmiR-141~200c, RT2_Zeb1*^*200H*^*_RipmiR-141~200c, RT2_Zeb1*^*200M*^*_RipmiR-141~200c, RT2_Zeb1*^*200H*^, *RT2_Zeb1*^*200M*^, *n* = 42, 8, 13, 8, 45, 26, respectively). **k** Blood glucose (*RT2*, *RT2_RipmiR-141~200c, RT2_Zeb1*^*200H*^*_RipmiR-141~200c, RT2_Zeb1*^*200M*^*_RipmiR-141~200c, RT2_Zeb1*^*200H*^, *RT2_Zeb1*^*200M*^, *n* = 11, 8, 9, 9, 11, 10, respectively). Dotted line represents onset of severe hypoglycemia. **l** Percent tumors per grade (*RT2*, *RT2_Zeb1*^*200M*^, *RT2_Zeb1*^*200H*^*_RipmiR-141~200c*, *RT2_Zeb1*^*200M*^*_RipmiR-141~200c,* from *n* = 5, 5, 8, 5 mice and *n* = 55, 16, 24, 15 tumors, respectively). **m** Percent insulin-positive and -negative tumors (*RT2, RT2_Zeb1*^*200M*^, *RT2_Zeb1*^*200H*^*_RipmiR-141~200c, RT2_Zeb1*^*200M*^*_RipmiR-141~200c*, from *n* = 5, 5, 8, 5 mice and *n* = 67, 15, 24, 15 tumors, respectively). **n** Percent animals with insulin-positive and -negative metastases (*RT2_Zeb1*^*200H*^, *RT2_Zeb1*^*200M*^, *RT2_Zeb1*^*200H*^*_RipmiR-141~200c*, *RT2_Zeb1*^*200M*^*_RipmiR-141~200c*, *n* = 7, 9, 8, 5, respectively). **o** Representative insulin and SV40 immunofluorescence of *RT2_Zeb1*^*200M*^_*RipmiR-141~200c* liver metastases (scale bar = 50 μm). **a**, **c**, **e**–**i** RNA sequencing of islets of 6-week-old mice (*RT2*, *RT2_DKO, RT2_Zeb1*^*200H*^, *RT2_Zeb1*^*200M*^, *n* = 2, 5, 3, 4, respectively). **d**, **k** Data are plotted as mean ± SD. Significance was evaluated by **a** empirical Bayes method, **d** one-way ANOVA with Dunnett’s multiple comparisons (vs. *RT2*), **e**, **f**, **h**, **i** competitive gene set test, **j** Mantel Cox test, and **k** two-tailed *t* test with Holm−Sidak correction (vs. *RT2*). *P ≤ 0.05; **P ≤ 0.01; ***P ≤ 0.001; ****P ≤ 0.0001

To investigate the *RT2_DKO* and *RT2_Zeb1*^*200M*^ phenotypes at the molecular level, we performed RNA sequencing of islets isolated from 6-week-old mice, to capture the primary changes in gene expression leading to the end-stage phenotype. Surprisingly, *Zeb1* transcript levels increased by only 30% in *RT2_DKO* and 55% in *RT2_Zeb1*^*200M*^ islets compared to *RT2* controls (Fig. 3a), a modest increase that was further confirmed at the protein level (Fig. 3b). Furthermore, comparison of wild-type and mutant Zeb1 allelic expression in *RT2_Zeb1*^*200H*^ islets revealed minor but consistently higher expression of the mutant allele compared to the *WT* allele (Fig. 3c). Zeb1 transcript and protein levels were evaluated in tumors as well, revealing an increase of 50, 37, and 105%, respectively, in protein levels in *RT2_DKO*, *RT2_Zeb1*^*200H*^ and *RT2_Zeb1*^*200M*^ animals compared to *RT2* (Fig. 3d; Supplementary Figure 3a, b).

We next explored whether this modest magnitude of Zeb1 derepression was sufficient to affect Zeb1 transcriptional networks downstream. Expression of potential Zeb1 target genes (from Zeb1 ChIP)^40^ was significantly downregulated in both *RT2_DKO* and *RT2_Zeb1*^*200M*^ islets relative to *RT2* controls (Fig. 3e, f). Interestingly, *Zeb1* target genes were significantly further downregulated in *RT2_Zeb1*^*200M*^ mice compared to *RT2_DKO* mice (Supplementary Figure 3c). Heat-map analysis also shows that Zeb1 target genes were highly differentially expressed in *RT2_Zeb1*^*200M*^ vs. *RT2* islets and more similar in expression to *RT2_DKO* islets (Fig. 3g). These results support the conclusion that it is indeed *Zeb1* and its target genes that mediate the common phenotype observed in *RT2_DKO* and *RT2_Zeb1*^*200M*^ mice, and that stronger regulation of Zeb1 in *RT2_Zeb1*^*200M*^ mice compared to *RT2_DKO* might contribute to the more extreme metastatic phenotype (Figs. 1l, 2l).

*ZEB1* and miR-200 expression inversely correlates in numerous cancer cell lines^41–43^. This expression pattern is due not only to miR-200-mediated regulation of *ZEB1* but also direct repression of both mir-200 clusters by ZEB1, establishing a double-negative feedback loop, which is thought to contribute to plasticity between the epithelial and mesenchymal states^20,24,25^. Measurements of miR-200 levels in *RT2_Zeb1*^*200M*^ islets revealed an 85−90% decrease of members of the miR-200a~200b~429 cluster and a 94% decrease in those of the miR-141~200c cluster relative to *RT2*, with no difference in expression levels in *RT2_Zeb1*^*200M*^ mice compared to *RT2_DKO* animals (Supplementary Figure 3d, e). This resulted in significant upregulation of predicted targets of the miR-200c family in *RT2_DKO* and *RT2_Zeb1*^*200M*^ islets (Fig. 3h, i), but surprisingly little increase in expression of miR-141 predicted targets (Supplementary Figure 3f, g). Of note, compared to ChIP targets of other random transcription factors, Zeb1 ChIP targets were not particularly enriched in predicted miR-200 targets (Supplementary Figure 3h), implying that in *RT2_Zeb1*^*200M*^ islets, miR-200c targets were regulated by miR-200c rather than directly by *Zeb1*.

To compensate for the loss of miR-200 in *Zeb1*-mutant mice, we crossed in a transgenic allele in which the expression of *MiR-141~200c* is controlled by the rat insulin promoter (Rip) and is thus independent of Zeb1 regulation (*RipmiR-141~200c*)^29^, thereby enabling stable expression of miR-141~200c despite elevated *Zeb1* expression (Supplementary Figure 3d, i). *RT2_Zeb1*^*200M*^_*RipmiR-141~200c* mice had reduced survival and blood glucose levels compared to *RT2_Zeb1*^*200M*^ and *RT2_Zeb1*^*200H*^ mice, presumably attributable to regulation of miR-200 targets other than *Zeb1*. Notably, survival and longitudinal blood glucose levels of *RT2_Zeb1*^*200H*^*_RipmiR-141~200c* mice were shifted towards *RT2* controls even further than the *RT2_Zeb1*^*200M*^*_RipmiR-141~200c* mice, indicating that the presence of one wild-type *Zeb1* allele was sufficient to partially rescue the phenotype in the presence of adequate levels of miR-141~200c (Fig. 3j, k). This partial rescue thus further highlighted the significance of tuning *Zeb1* expression in the process of dedifferentiation and invasion of islet-cell carcinomas. Furthermore, while the expression of the *RipmiR-141~200c* allele neither reduced the invasiveness of primary tumors (Fig. 3l) nor inhibited downregulation of Cdh1 (Supplementary Figure 3j), these tumors were more frequently insulin-positive than *RT2_Zeb1*^*200M*^ tumors (Fig. 3m), thus explaining the shortened survival and lower blood glucose upon miR-141/200c re-expression. In addition, while metastases in *RT2_DKO, RT2_Zeb1*^*200H*^, and *RT2_Zeb1*^*200M*^ mice were insulin-negative, *RT2_Zeb1*^*200H*^*_RipmiR-141~200c* and *RT2_Zeb1*^*200M*^*_RipmiR-141~200c* mice had insulin-positive metastases (Fig. 3n, o; Supplementary Figures 1e, 2h). Taken together, these data suggest that while direct Zeb1 targets are responsible for the invasive phenotype, Zeb1’s influence on dedifferentiation is mediated at least in part via its downregulation of miR-200.

Finally, we sought to determine the relative importance of *Zeb1* vs. other miR-200 targets in tumor progression. As predicted miR-141 targets were not significantly regulated in our model (Supplementary Figure 3f, g), we selected the most upregulated miR-200c predicted targets in *RT2_DKO* vs. *RT2* and in *RT2_Zeb1*^*200M*^ vs. *RT2* islets (of which 80.2% were overlapping) and performed an siRNA screen in an *RT2_DKO* tumor-derived cell line. As EMT is a well-described mechanism of metastasis development^44^ and thought to be regulated by miR-200 based on multiple in vitro and correlative studies^18,45^, we specifically sought to understand the relative role of these predicted targets in repressing the epithelial phenotype in *RT2_DKO* and *RT2_Zeb1*^*200M*^ tumors by measuring mRNAs of epithelial markers, E-Cadherin (*Cdh1*), Epithelial Cell Adhesion Marker (*Epcam*), and Occludin (*Ocln*) upon siRNA knockdown. Knockdown of *Zeb1* was by far the most efficient at inducing both *Cdh1* (Supplementary Figure 3k) and *Epcam* (Supplementary Figure 3l), and among the most efficient at inducing *Ocln* (Supplementary Figure 3m), supporting the hypothesis that the phenotype in *RT2_DKO* mice was predominantly mediated through ZEB1 and its downstream regulation of EMT.

### miR-200 regulation of *Zeb1* engages the EMT program in vivo

**Fig. 4:**
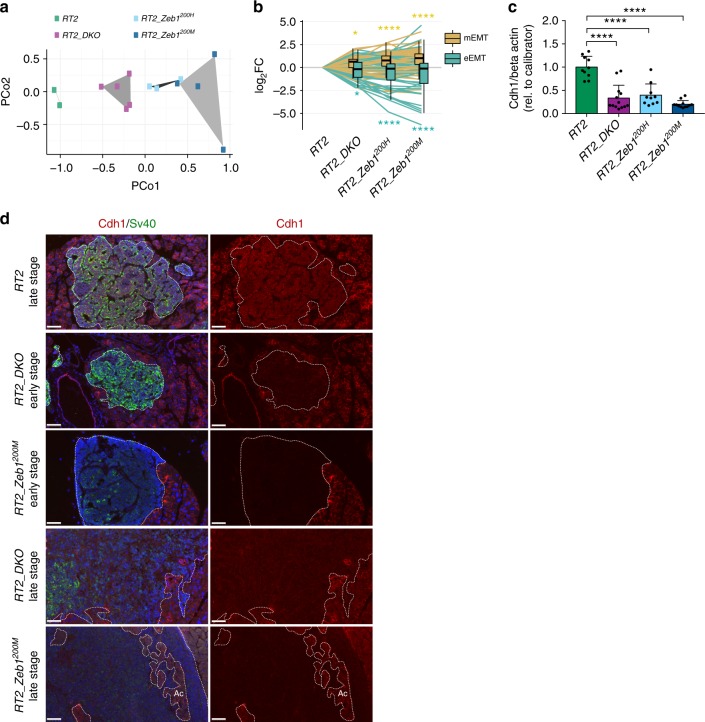
miR-200-mediated *Zeb1* regulation engages EMT program in vivo. **a** Principal coordinate analysis based on the expression of 122 EMT core genes in islets of 6-week-old mice. **b** Box-and-whisker plots (box, 25th and 75th percentiles; central line, median) of log_2_FC of mEMT or eEMT mRNAs in *RT2_DKO, RT2_Zeb1*^*200H*^, and *RT2_Zeb1*^*200M*^ relative to *RT2*. **c** CDH1 quantification in end-stage tumors (immunoblots shown in Supplementary Figure 3b) (*n* = 10–13 tumors), normalized to β-actin and to calibrator sample. Data represent mean + SD. **d** Representative Cdh1 and SV40 immunofluorescence stainings of tumors (scale bar = 50 μm). Tumors are outlined with dotted lines. Ac, Acinar cells. **a**, **b** RNA sequencing of islets of 6-week-old mice (*RT2*, *RT2_DKO*, *RT2_Zeb1*^*200H*^, *RT2_Zeb1*^*200M*^, *n* = 2, 5, 3, 4 mice, respectively). Significance was evaluated by **b** competitive gene set test and **c** one-way ANOVA followed by Dunnett’s multiple comparisons test (vs. *RT2*). *P ≤ 0.05; ****P ≤ 0.0001

We next sought to determine whether we could detect broader EMT transcriptional changes in *RT2_DKO* and *RT2_Zeb1*^*200*^ islets at the pretumor stage. Interestingly, Ingenuity pathway analysis of differentially expressed genes revealed enrichment in functions including cellular movement, organismal development, cell-to-cell signaling and interaction, and cell morphology, which are key aspects of the EMT process (Supplementary Figure 4a). Further analysis was performed utilizing a list of core EMT genes generated from a meta-analysis of 18 independent studies^46^. Principal coordinate analysis revealed a gradient of clusters that grouped according to genotype and phenotype, with the *RT2_DKO* mice exhibiting the less severe metastatic phenotype, located between the *RT2* control mice and the more extreme *RT2_Zeb1*^*200M*^ mice (Fig. 4a). The EMT process involves complex gene-expression changes; EMT genes can be subdivided into genes that are downregulated, i.e. associated with the epithelial phenotype (eEMT), or upregulated, i.e. associated with the mesenchymal phenotype (mEMT). eEMT and mEMT mRNA levels were significantly regulated in both *RT2_DKO* and *RT2_Zeb1*^*200M*^ animals, with greater fold-change regulation in *RT2_Zeb1*^*200H*^ and *RT2_Zeb1*^*200M*^ islets in general (Fig. 4b), consistent with greater regulation of *Zeb1* and its targets in these animals. Specifically, classic mEMT markers such as *Vim*, *Twist1* and *Snai1* were consistently increased in all three metastatic models, and levels of eEMT markers *Ocln*, *Epcam*, and *Cdh1* were strongly reduced (Supplementary Figure 4b). Furthermore, Cdh1 protein, whose loss is a key contributor to EMT, tumor malignancy, and progression^47,48^, was also substantially downregulated in islets and tumors (Figs. 3b, 4c, d; Supplementary Figure 3a, b). Of note, analysis of liver metastases in both *RT2_DKO* and *RT2_Zeb1*^*200M*^ mice revealed no re-expression of Cdh1, suggesting that in this model, mesenchymal-to-epithelial transition (MET) is unlikely to be required for metastatic growth (Supplementary Figure 4c). Finally, the stem-cell markers *Sox2*, *Tcf4*, and *c-myc* were upregulated in *RT2_Zeb1*^*200M*^ islets, in line with the acquisition of stemness properties, which has been associated with EMT^49,50^ (Supplementary Figure 4b).

### *Zeb1* and EMT are regulated by miR-200c but not by miR-141

**Fig. 5:**
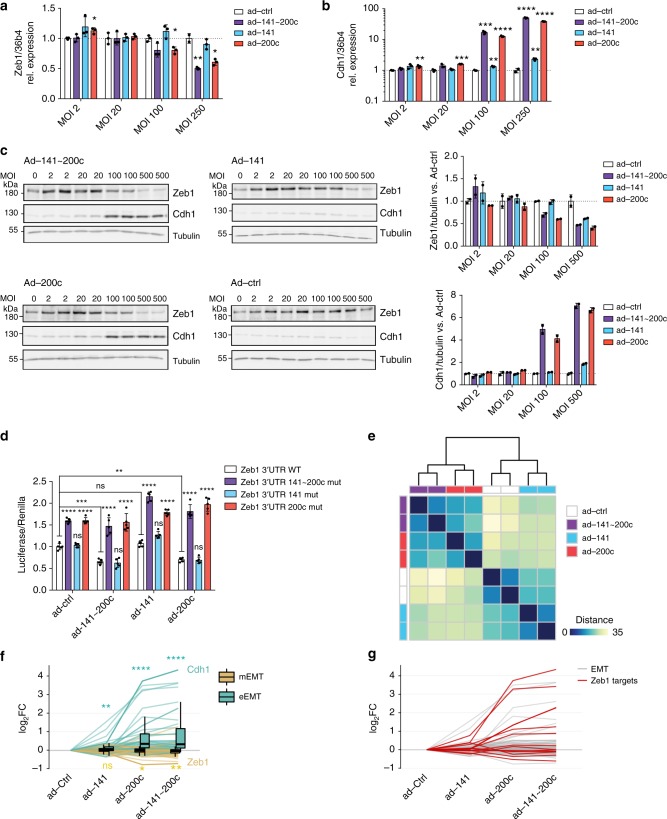
Zeb1 and EMT programs are regulated by miR-200c but not by miR-141. **a**, **b** Relative *Zeb1* (**a**) and *Cdh1* (**b**) expression in *RT2-DKO*-derived cells infected with recombinant adenoviruses expressing miR-141, -200c, or both at the indicated MOIs (*n* = 3 replicates). Expression was measured by qPCR and normalized to Ad-Ctrl at the corresponding MOI. **c** Immunoblots depicting ZEB1 and CDH1 expression in lysates of *RT2_DKO*-derived cells infected with the indicated adenoviruses at the specified MOIs. Band densities were quantified and normalized to tubulin and to appropriate ad-Ctrl (right-hand graphs). **d** Normalized luciferase assays (*n* = 6 replicates) for WT *Zeb1* 3′UTR, *Zeb1*^*200*^ 3′UTR, and *Zeb1* 3′UTRs harboring mutations either in miR-141 or miR-200c sites, performed in Min6 cells with endogenous or adenoviral overexpression of miR-200 (MOI 50). **e** Hierarchical clustering and heat-map analysis of the top 500 genes with the highest biological coefficient of variation in *RT2_DKO* cells infected with ad-141~200c, ad-141, or ad-200c relative to ad-Ctrl-infected cells. The scale bar indicates the Euclidian distance between samples. **f** Box-and-whisker plots (box, 25th and 75th percentiles; central line, median) of log_2_FC of mEMT and eEMT genes in *RT2_DKO* cells infected with ad-141~200c, ad-141, or ad-200c relative to ad-Ctrl-infected cells. **g** Overlap between Zeb1 ChIP targets and EMT genes. Colored are the results for EMT genes that are also Zeb1 ChIP targets. **e**−**g** RNA sequencing of adenovirus-infected *RT2_DKO* cells (*n* = 2 technical replicates for each adenoviral infection). **a**–**d** Data are plotted as mean ± SD. Significance was evaluated by **b** multiple two-tailed *t* tests (vs. respective Ad-Ctrl) with Holm−Sidak correction, **d** two-way ANOVA with Tukey’s multiple comparisons test (vs. respective Zeb1 3′UTR WT and vs. Ad-Ctrl/ WT Zeb1 3′UTR as indicated), and **f** competitive gene set test. *P ≤ 0.05; **P ≤ 0.01; ***P ≤ 0.001; ****P ≤ 0.0001

To distinguish the specific roles of the miR-200c and miR-141 families, we infected *RT2_DKO* tumor-derived cells with adenoviruses harboring either the endogenous *Mir141~200c* locus (ad-141~200c) or the *Mir141* or *Mir200c* genes alone (ad-141 and ad-200c, respectively) (Supplementary Figure 5a, b). Overexpression of miR-200c led to a dosage-dependent repression of *Zeb1* transcripts and strong induction of *Cdh1* mRNA, which was not observed upon miR-141 overexpression (Fig. 5a, b). Similar regulation was also evident at the protein level, with a response to miR-141 expression observed only at MOI 500, a titer at which some cell toxicity was noted (Fig. 5c). In contrast, forced overexpression of miR-200c in a *RT2_Zeb1*^*200M*^ cell line did not change Zeb1 or Cdh1 levels (Supplementary Figure 5c–g). In addition, *Zeb1* 3′UTR luciferase assays in the *Min6* beta-cell line, which endogenously expresses both miR-141 and miR-200c, revealed that mutations of miR-141 sites alone did not cause derepression compared to the WT 3′UTR, whereas 3′UTRs harboring mutations in only miR-200c sites or in both miR-141 and miR-200c sites were derepressed similarly (Fig. 5d). Moreover, miR-141 overexpression had no effect on the reporter harboring the WT 3′UTR, whereas overexpression of miR-200c or miR-141~200c led to decreased reporter activity (Fig. 5d). These results demonstrate that members of the miR-200c family, but not of the miR-141 family, function as major regulators of *Zeb1* and its downstream network.

To delineate the roles of the miR-141 and miR-200c families at regulating EMT more broadly, we performed RNA sequencing of *RT2-DKO*-derived cells infected with adenovirus to achieve miR-141 and miR-200c expression levels similar to endogenous levels in *RT2* islets (Supplementary Figures 3e, 6a). We confirmed that predicted miR-200 targets were only regulated upon expression of their respective seed family (Supplementary Figure 6b). Hierarchical clustering analysis revealed that ad-141~200c- and ad-200c-infected cells clustered together, whereas ad-141-infected cells were more similar to ad-Ctrl-infected cells (Fig. 5e). Ingenuity analysis identified cellular movement as the most regulated functional category upon miR-141~200c or miR-200c expression (Supplementary Figure 6c), consistent with a major role in EMT regulation. Similar analysis for ad-141-infected cells revealed no enrichment in any functional annotation, due to the small number of significantly regulated genes. Although eEMT genes were significantly upregulated in ad-141-infected cells (*P* = 2.52e^−03^), the fold-change was much greater in ad-200c- and ad-141~200c-infected cells (*P* = 6.52e^−41^ and *P* = 4.99e^−45^, respectively), with only a small additional increase in fold-change of ad-141~200c- compared to ad-200c-infected cells. Similarly, mEMT genes were not significantly downregulated upon miR-141 expression, but were reduced upon overexpression of miR-200c (*P* = 2.78e^−02^) and miR-141~200c (*P* = 8.49e^−03^) (*P* values calculated by hypergeometric test) (Fig. 5f). Collectively, these results indicate that miR-200c, but not miR-141, profoundly inhibits the process of EMT, with the strongest effect of miR-200c treatment being derepression of eEMT genes.

Mechanistically, this effect was indirect, as miR-200c predicted targets significantly overlapped with mEMT genes (*P* = 7.6e^−03^) but not with eEMT transcripts (*P* = 2.3e^−01^) (Fig. 5f; Supplementary Figure 6d), suggesting that miR-200c acts by repressing mEMT genes, which in turn upregulates eEMT genes. Indeed, the most strongly repressed mEMT gene was *Zeb1* (Fig. 5f), with significant overlap between Zeb1 ChIP targets and eEMT transcripts (*P* = 2e^−10^) (*P* values calculated by hypergeometric test) (Fig. 5g, Supplementary Figure 6e). These results suggest that it is primarily miR-200c, and not miR-141, that represses mesenchymal genes including *Zeb1*, thereby enabling maintenance of epithelial markers such as *Cdh1* that are crucial for preventing EMT-induced tumor progression.

## Discussion

The miR-200–Zeb1 axis has been extensively studied in cancer and implicated in tumor progression through expression analysis of clinical specimens^18,20,26–28^. Although most correlation studies associate loss of miR-200 with increased tumor progression^18^, gain of miR-200 has also been associated with disease progression^51–53^, thus emphasizing the need for genetic studies to better understand miR-200 mechanisms in various tumor contexts. Reduction and overexpression of miR-200 in vitro can be sufficient to induce or reverse EMT, respectively, and Zeb1 is postulated to be important in this process^41^. Independent manipulation of Zeb1 expression strongly affects EMT in vitro^54^, and a recent study demonstrated that in vivo ablation of *Zeb1* in PDAC led to decreased tumor progression and metastasis^16^. Although these studies indicate that the miR-200–Zeb1 axis is important in EMT, they do not address whether in vivo regulation of Zeb1 to the extent achieved by miR-200 is sufficient to have phenotypic consequence. This question can be addressed by mutating the miR-200 sites in the *Zeb1* 3′UTR, which we show here in two epithelial cancer models to be sufficient to induce EMT and have strong effects on tumor dedifferentiation, progression, and invasion.

Previous work in the *RT2* insulinoma model has revealed that tumor progression occurs with distinct mRNA and miRNA expression profiles at each stage^34,55^. Interestingly, it was observed that miR-200 levels were decreased and Zeb1 expression increased in advanced stage tumors, suggesting that *RT2* tumors endogenously regulate the miR-200–Zeb1 axis^55^. In addition, sustained Cdh1 expression was shown to be important in preventing the transition from adenoma to carcinoma^56^, further emphasizing the role of EMT in tumor progression in the *RT2* model. The presence of rare low-insulin, “met-like primary” tumors in the *RT2* model has also been reported^34^, reminiscent of the dedifferentiated invasive tumors that we identified in *RT2_DKO* and *RT2_Zeb1*^*200M*^ mice. While the authors suggest that these tumors may derive either from differentiated tumors or directly from islet progenitor cells, gene-expression data in *RT2_DKO* and *RT2_Zeb1*^*200M*^ mice suggest an EMT-associated dedifferentiation program, as expression of mature β-cell markers is reduced while β-cell progenitor markers are not consistently expressed.

In this study, we selected the *RT2* beta-cell tumor model because previous work in beta cells had demonstrated that miR-200c is a potent regulator of antiapoptotic genes and that miR-200c knockout is thus protective against apoptosis^29^. Our data reveal a decrease in apoptosis in *RT2_DKO* and *RT2_Zeb1*^*200M*^ islets and tumors relative to *RT2*, suggesting a similar role for miR-200 in beta-cell tumors^29^. Furthermore, our results thus suggest that modest derepression of Zeb1 is sufficient to increase resistance to apoptosis, a common feature in EMT^6,50^. This function might be a direct effect of Zeb1 on its targets, or an indirect effect through its downregulation of miR-200 through the miR-200–Zeb1 double-negative feedback loop.

miR-200 and Zeb1 are involved in a double-negative feedback loop that is thought to stabilize either the epithelial or mesenchymal state, as well as permit a high degree of plasticity between the two states^20,24,25^. Our data demonstrate the in vivo impact of this loop. Expression analysis of the miR-200 family showed that all five miRNAs were downregulated by ~90% in *RT2_Zeb1*^*200M*^ islets, similar to the degree of regulation previously reported in vitro^25^. This downregulation had molecular consequences, reflected by strong enrichment in miR-200c target regulation, and phenotypic consequences, as re-expression of miR-141~200c in *RT2_Zeb1*^*200M*^ mice reduced their survival and accelerated hypoglycemia. This demonstrates that part of Zeb1’s mechanism of action is through its regulation of miR-200 and its targets, and that these miR-200c targets play a particularly strong role in dedifferentiation. It should be emphasized, however, that in our analyses, Zeb1 targets identified by ChIP significantly overlapped with EMT genes and were strongly regulated, indicating that Zeb1 also acts in an miRNA-independent manner, as exemplified by its direct regulation of *Cdh1* and *Epcam*.

To our surprise, *RT2_Zeb1*^*200M*^ mice exhibited a stronger and more penetrant phenotype than *RT2_DKO* animals, with tumors that were more invasive and more frequently insulin-negative, and a higher penetrance of macrometastasis. Although seemingly counterintuitive, the observation that miR-200 regulation of a single target gene had a stronger effect than knockout of the entire miR-200 family could be due to differential regulation of other miRNA targets that might be protective against tumor progression, as the negative feedback loop in *RT2_Zeb1*^*200M*^ tumors did not completely ablate miR-200 levels. Another possibility relates not to the primary tumor but to metastatic growth: perhaps some plasticity of miR-200 regulation is required for efficient seeding and metastasis development, which would have been precluded in the tumor cells of the miR-200-null *RT2_DKO* model.

The miR-200 superfamily is often studied as a single family, but is expressed from two independent polycistronic genomic loci harboring two separate families, which complicates the functional analysis of the different families. Our results demonstrate greater importance for the *Mir141~200c* cluster in regulating dedifferentiation and invasion of *RT2* tumors than the *Mir200a~200b~429* cluster. This could be attributable to the lower expression of the latter cluster in islets^29^, which may be tissue-dependent. Our results also show that the miR-200c family was more potent in regulating Zeb1 and EMT than the miR-141 family. This we observed not only in vitro, consistent with results of several other studies^24,41^, but also in vivo, as the EMT transcriptional changes that occurred in *RT2_DKO* and *RT2_Zeb1*^*200M*^ islets were accompanied by a response of miR-200c but not miR-141 predicted targets. Interestingly, absolute quantification of miR-200c and miR-141 revealed 2.5-fold lower expression of miR-141 in islets, although whether this was an accurate measurement of miRNA levels is unclear due to a potential bias against miRNAs with low GC content during Trizol extraction^57^. To ensure that the lack of Zeb1 response was not simply due to insufficient miR-141 levels, we performed luciferase assays and found no response of the Zeb1 3′UTR upon miR-141 site mutation and miR-141 overexpression. In agreement with our findings, in a xenograft model, miR-141-overexpressing tumor cells have a greater capacity to colonize lungs than miR-200c-overexpressing cells^58^.

The EMT program is regulated by a complex network of transcription factors including the SNAIL, TWIST, and ZEB families, whose expression and importance in EMT is tissue-dependent^13,16^. In our system, Zeb1 upregulation was sufficient to initiate and drive the EMT program. Key EMT-TFs *Zeb2*, *Snai1*, and *Twist1* were upregulated as well, but *Twist1* was lowly expressed and unlikely to play a major role. In development and cancer, Snai1 expression typically precedes and helps induce the expression of other EMT TFs including Zeb1^59–61^, and thus it is interesting that in our system Zeb1 can induce Snai1, perhaps through indirect mechanisms. Although *Cdh2* was not increased in *RT2_DKO* and *RT2_Zeb1*^*200M*^ islets, *Vim*, a marker for end-stage EMT^62^, was strongly upregulated in *RT2_DKO* and *RT2_Zeb1*^*200M*^ islets. Furthermore, the strong induction of stem-cell marker *Sox2* along with induction of *Tcf4*, *c-myc*, and *Lgr5* was in line with the dedifferentiation and acquisition of stemness properties associated with EMT and regulated by Zeb1^49,50^. Importantly, these molecular signatures of EMT were already detected in islets of 6-week-old mice, before tumor development, suggesting that they were all driven by miR-200 loss and Zeb1 overexpression and not by secondary mutations.

An important conclusion of this study is that only subtle changes in Zeb1 expression, to the degree conferred by dosage-dependent miR-200c regulation, are sufficient to induce a strong effect on EMT and cancer progression in two tumor-susceptible genetic backgrounds, the RT2 and KPC models. This is further highlighted by the observation that miR-200c-mediated repression of a single wild-type allele in *RT2_Zeb1*^*200H*^*_RipmiR-141~200c* mice is sufficient to partially rescue the phenotype relative to *RT2_Zeb1*^*200M*^*_RipmiR-141~200c* mice. In this regard, it would be interesting to investigate the regulation of miR-200, Zeb1 and their targets in the progression of human insulinomas to metastatic, nonfunctional pancreatic neuroendocrine tumors (PanNET). Given the importance of the miR-200–Zeb1 axis in many human cancers^20,49,51^, this suggests the potential therapeutic utility of enhancing miR-200c or repressing Zeb1 in primary tumors. This is especially compelling because even small reductions of Zeb1 levels could have considerable impact in preventing EMT and curtailing metastasis, which in turn could have a profound impact in reducing cancer progression and mortality. Finally, manipulation of the miR-200–Zeb1 axis could also be relevant in noncancer settings: beta-cell dedifferentiation has been shown to be a hallmark beta-cell defect in type 2 diabetes; therefore, reversing this process could be a novel therapeutic strategy^63–66^. It would thus be important to study the miR-200–Zeb1 axis in nontumor sensitized diabetic models. A further application could be to improve differentiation of beta-cells derived from ES cells or to counter the dedifferentiation of transplanted islets and their in vitro culture, all major hurdles for beta-cell transplantation therapy^67–69^.

## Methods

### Experimental animals

Mice were housed in a pathogen-free animal facility at the Institute of Molecular Health Sciences at the ETH Zurich. The animals were maintained in a temperature-controlled room (22 °C), with humidity at 55% and on a 12 h light−dark cycle (lights on from 0600 to 1800 hours). Mice were fed a standard laboratory chow and water ad libitum, and the age of mice is indicated in the figures and text. All ethical regulations were complied with and all animal experiments were approved by the Kantonale Veterinäramt Zürich. Animals in the RT2 background were in a C57BL/6 background. The generation of mice deficient for *Mir141~200c* and *Mir200a~200b~429* was described previously^29^. *Rip-*Cre mice were kindly provided by P. Herrera and *Rip-Tag2* mice were provided by D. Hanahan. The generation and breeding schemes of *Rip-Tag2* mice have been published^30^; end-stage was determined as the time point at which random-fed blood glucose remained below 2.0 mmol/L for 3 consecutive days or dipped below 1.0 mmol/L on any single day, or in the case of *RT2_DKO* or *RT2_Zeb1*^*200M*^ mice whose blood glucose never went below 2.0 mmol/L, based upon general termination criteria including ruffled fur, hunched posture, and loss of the escape reflex. The LSL-Kras^G12D/+^; LSL-Trp53^R172H^; LSL-Trp53^R17H/+^ (*KPC*) mouse model of pancreatic adenocarcinoma was in a mixed 129/Sv BALB/C C57BL/6 background, and all experiments involved littermate controls; end-stage was determined as the timepoint at which mice lost 10% of their body weight.

### Generation of *Zeb1*^*200*^ mutant mice

To generate the targeting construct, a DNA fragment containing the mouse *Zeb1* locus was subcloned from bacterial artificial chromosome (BAC RP23-78D21). The targeting arms spanned exon 8 of Zeb1 including its entire 3′UTR. All nine miR-200 sites were mutated by site-directed mutagenesis. A LoxP-flanked puromycin-resistance gene was used for positive selection and diphtheria toxin gene was used for negative selection. Thirty-three positive clones were obtained from 246 colonies of which two were injected into C57BL/6j blastocysts and transferred into pseudo-pregnant females. Chimeras were bred with C57BL/6j mice to generate heterozygous progeny and germline transmission of the mutated allele was verified by PCR. The puromycin-resistance cassette was excised by intercrossing with transgenic Cre deleter mice. The phenotype of *RT2_Zeb1*^*200H*^ and *RT2_Zeb1*^*200M*^ mice was confirmed in mouse lines derived from the two independent ES cell clones.

### Blood-glucose measurements

Blood glucose was measured using a Contour glucometer (Bayer).

### Cell line generation from *RT2_DKO* and *RT2_Zeb1*^*200M*^ tumors

To generate tumor-derived cell lines, tumors were isolated from *RT2_DKO* and *RT2_Zeb1*^*200M*^ mice, minced, and digested for 18 min in Liberase TM (Roche) at 37 °C, then plated in Primaria (Corning) plates. Media were changed daily to remove dead cells and debris, and cells were passaged after 5–7 days and replated. Experiments were conducted with cells at ~P20.

### Immunohistochemistry, lesion staging, and image analysis

Pancreata and livers were fixed in 4% paraformaldehyde, embedded in paraffin, and cut into 3.5 μm sections. Antigen retrieval was performed with 10 mM sodium citrate buffer (pH 6.0). Sections were permeabilized and blocked in phosphate buffered saline (PBS) containing 0.1% Triton-X-100, 1% bovine serum albumin (BSA) and 5% donkey or goat serum. Primary antibody binding was performed overnight at 4 °C, while secondary antibody incubation was carried out at room temperature for 1 h. Tissue samples were stained with hematoxylin and eosin (H&E) according to routine laboratory procedures. Slides were scanned using a ×20 objective on the Pannoramic 250 Slide scanner (3D Histech) or a ×40 objective on the Aperio AT2 Slide scanner (Leica Biosystems). For experiments in the *RT2* background, mice were tail-vein injected with 10 mg/g body weight of EdU (ThermoFisher) and sacrificed 2 h later. EdU incorporation in islets and tumors was analyzed using the Click-iT EdU Alexa Fluor 647 HCS Assay (Invitrogen) kit, and the ratio of EdU-positive nuclei to total nuclei was analyzed for each lesion using QuPath^70^. Similarly, TdT-mediated dUTP-biotin nick end labeling (TUNEL) staining was performed using the ApopTag Red In Situ Apoptosis Detection Kit (Merck) and the ratio of TUNEL-positive to total nuclei was also quantified for each lesion using QuPath. For islet and tumor staging, all lesions were analyzed blindly by a Board-certified pathologist in one representative H&E section of five mice per age and genotype and classified according to the guidelines provided in Lopez and Hanahan^35^. Vascular and lymphatic invasions were defined as the presence of SV40-positive cells within a CD31-expressing vessel, and total instances were counted in five representative sections per age and genotype. For analysis of the KPC model, degree of metastasis was determined by calculating the percentage of CK19-positive area over the total DAPI-surface area using NIH ImageJ software (https://imagej.nih.gov/ij/). KPC tumors were graded according to the recommendations presented in the consensus report by Hruban et al.^71^

### Tumor burden and macrometastasis penetrance

Tumors were microdissected from pancreata of mice at various ages and measured in three dimensions. Individual tumor volume was approximated using the formula length × width^2^ × 0.52, and total tumor burden was calculated as the sum of volumes of all tumors in one mouse pancreas. Macrometastatic penetrance was determined based on the presence of metastases visible without the use of a microscope on the liver or on the intestine.

### Immunoblotting

Small tumors, pieces of tumors, or pooled islets (2 mice per lane) were disrupted in RIPA buffer (150 nM NaCl, 1% Triton-X, 50 mM Tris, 0.5% sodium deoxycholate, 0.1% SDS), supplemented with Complete, ethylenediaminetetraacetic acid (EDTA)-free Protease inhibitors (Roche) and Halt Phosphatase inhibitors (Pierce) using the TissueLyser (Qiagen), followed by sonication. Cells were washed in PBS, then collected in RIPA buffer and sonicated directly. Protein concentrations were measured by bicinchoninic acid assay. Laemmli buffer was added to samples, equal protein amounts were separated by sodium dodecyl sulfate–polyacrylamide gel electrophoresis (SDS-PAGE), transferred by electroblotting and membranes blocked in 5% milk/Tris buffered saline with Tween 20 (TBS-T) for 1 h. Membranes were incubated with appropriate antibodies overnight at 4 °C, then exposed to secondary antibodies for 1 h at room temperature and developed using ECL Western Blotting Substrate. Band density was evaluated using MultiGauge (Fujifilm) and normalized to beta actin or tubulin levels. For blots intended to be compared to each other, data were additionally normalized to a calibrator sample that was equally loaded on each blot. Uncropped western blots can be found in Supplementary Figures 7–10.

### Antibodies

The following antibodies were used in immunoblotting: anti-E-cadherin (Cell Signaling, 3195, 1:1000), anti-Zeb1 (Santa Cruz, sc-25388, 1:1000), anti-Beta-Actin (Cell Signaling, 4970S, 1:1000), and anti-Gamma-Tubulin (Sigma, T6557, 1:5000). The following antibodies were used for immunohistochemistry: anti-SV40 T Ag (Santa Cruz, sc-20800, 1:50), anti-Insulin (DAKO, A056401, 1:1000), anti-CD31 (R&D Systems, AF3628, 1:15), anti-Lyve1 (Abcam, ab14917, 1:100), anti-E-cadherin (Abcam, ab76055, 1:200), anti-Glucagon (Millipore, AB932, 1:200), anti-SMA (Abcam, ab5694, 1:100), anti-CK19 (Abcam, ab52625, 1:400).

### Recombinant adenoviruses

Recombinant adenovirus-expressing *pre-mir-141~200c* was previously generated^29^. In brief, 450 bp- and 250 bp-spanning sequences of pre-miR-141~200c were cloned into pcDNA3 and pAd5 for adenovirus production (Viraquest). Adenoviruses expressing miR-141 or miR-200c were generated by PCR amplification of pre-mir-141 or pre-mir-200c with ~200 bp flanking regions from mouse gDNA using primers listed in the Supplementary Table 1. Fragments were cloned downstream of a CMV promoter in pVQAd CMV K-NpA for recombinant adenovirus production (ViraQuest). All adenoviruses expressed *GFP* from an independent promoter. Ad-Ctrl was based on the same vector backbone (including GFP) but lacked the miRNA transgenes. Cell experiments with *RT2-DKO-* or *RT2-Zeb1*^*200M*^-tumor-derived cell lines were performed in a 24-well (qPCR) or 6-well format (Immunoblotting), by infecting cells 12 h post-seeding, and harvesting 48 h later. Data were normalized to the Ad-Ctrl-treated samples with the equivalent MOI. For the sequencing experiment, *RT2_DKO* cells were infected at MOI 100 and harvested 48 h later.

### Luciferase assays

The WT *Zeb1* 3′UTR was PCR-amplified from cDNA of Min6 cells (gift from C. Wollheim, Geneva), and the *Zeb1* 3′UTR harboring miR-200 site mutations was PCR-amplified from a *RT2_Zeb1*^*200M*^ tumor-derived cell line (primers listed in Supplementary Table 1) and cloned into pmirGLO. *Zeb1* 3′UTRs with either miR-200c or miR-141 sites mutated were de novo synthesized (Genscript) and cloned into pmirGLO. Min6 cells were cultured in 96-well plates and transfected with 400 ng of pmirGLO reporters. For luciferase experiments involving adenoviral overexpression (MOI 50) of miR-141~200c, miR-141, or miR-200c, cells were infected upon media change 6 h post-transfection with the pmirGLO plasmids. Cells were harvested and assayed 48 h after transfection using the Dual-Luciferase Reporter Assay System (Promega). Results were normalized to the Renilla luciferase control contained in pmirGLO and expressed relative to ad-Ctrl-treated cells transfected with the reporter containing the WT *Zeb1* 3′UTR.

### miRNA and gene-expression analysis

RNA was extracted using TRI Reagent (Sigma-Aldrich). miRNA expression analysis was performed using TaqMan MicroRNA Assays (ThermoFisher). For gene-expression analysis, RNA was reverse transcribed using the High Capacity cDNA reverse transcription kit (ThermoFisher), and qPCR was conducted using the gene-specific primers listed in Supplementary Table 1 and 2x KAPA SYBR FAST qPCR MM (Kapa Biosystems). Relative expression values were calculated using the ddCT and Pfaffl methods employing snoRNA202 for miRNA or mouse *36b4* (*Rplp0*) for gene-expression normalization. For absolute quantification of miRNA levels and the ability to compare between different TaqMan miRNA probes, synthetic miRNAs (Microsynth) comprising the mature miRNA sequence (mmu-miR-200c-3p, 5′-UAAUACUGCCGGGUAAUGAUGGA-3′; mmu-miR-141-3p, 5′-UAACACUGUCUGGUAAAGAUGG-3′) were serially diluted and spiked into *RT2_DKO* RNA (no endogenous miR-200 expression) and used to build a standard curve.

### siRNA knockdown

For the siRNA screen, a list of regulated miR-200c target genes was generated by selecting predicted miR-200c target genes (Targetscan v6.2) that were significantly (FDR ≤ 0.05) and minimum 1.3-fold upregulated in an initial set of RNA sequencing samples of 6-week-islets. *RT2_DKO* cells were seeded in 24-well plates and transfected the next day with 50 nM siRNA (pools of four siRNAs, Dharmacon, Supplementary Table 1) using RNAiMax (ThermoFisher). Cells were harvested 48 h later, and RNA was extracted for subsequent RT-qPCR. Data were normalized to results from cells transfected with pooled siRNAs to a scrambled control. To further confirm our results using Zeb1 siRNAs and discount the possibility of off-target effects, we performed deconvolution of our siRNA pools by transfecting cells with individual siRNAs and confirmed consistent regulation of Cdh1, Epcam, and Ocln upon Zeb1 knockdown.

To confirm the identity of the correct ZEB1 band in immunoblots with *RT2_DKO* cells as well as the specificity of knockdown of Zeb1 rather than Zeb2 (Supplementary Figure 9b), *RT2_DKO* cells were transfected with 50 nM siZeb1, siZeb2, or a combination of both, and harvested 72 h later.

### RNA sequencing

RNA was extracted from islets isolated from 6-week-old mice using the Picopure RNA Isolation kit (ThermoFisher), and from cell lines using TRI-Reagent (Sigma-Aldrich) followed by RNeasy column purification (Qiagen), with on-column DNase (Qiagen) digestion for all samples. Library preparation using the TruSeq Stranded mRNA Library Prep Kit (Illumina) and sequencing was performed at the Functional Genomics Center Zurich (FGCZ) on a HiSeq 4000 platform.

### Statistical analysis

Numerical values are reported as average ± s.d. unless stated otherwise. No statistical method was used to predetermine sample size; sample size was instead based on preliminary data and previous publications as well as observed effect sizes. No randomization of animals was performed, but animals were age-matched, and littermates were used whenever possible. Statistical analysis was performed using GraphPad Prism 7.0. If not mentioned otherwise in the figure legend, statistical significance (*P ≤ 0.05; **P ≤ 0.01; ***P ≤ 0.001, ****P ≤ 0.001) was determined by unpaired two-tailed *t* test, or one-way Anova with relevant post-hoc tests (Dunnett if not specified otherwise). *P* values for pathway enrichment were determined by Ingenuity software (Qiagen).

### Bioinformatic analysis of sequencing data

Detailed information on bioinformatics analysis of sequencing data can be found in the Supplementary Methods.

## Electronic supplementary material

Supplementary Information

## Data Availability

Sequencing data are available in ArrayExpress under accession number E-MTAB-6717.
