# Supplementary material for: Genetic dissection of the miR-200–Zeb1 axis reveals its importance in tumor differentiation and invasion
Source: Nat Commun. 2018 Nov 7;9:4671. doi: 10.1038/s41467-018-07130-z (PMC6220299; doi:10.1038/s41467-018-07130-z)
Supplement: Supplementary file 1 — Supplementary Information [file 41467_2018_7130_MOESM1_ESM.pdf]

Supplementary Information

Supplementary Figures 1–10, Supplementary Table, Supplementary Methods and Supplementary References

Title:

Genetic dissection of the miR-200–Zeb1 axis reveals its importance in tumor differentiation and invasion

Alexandra C. Title, Sue-Jean Hong, Nuno D. Pires, Lynn Hasenöhrl, Svenja Godbersen, Nadine Stokar-Regenscheit, David P. Bartel and Markus Stoffel

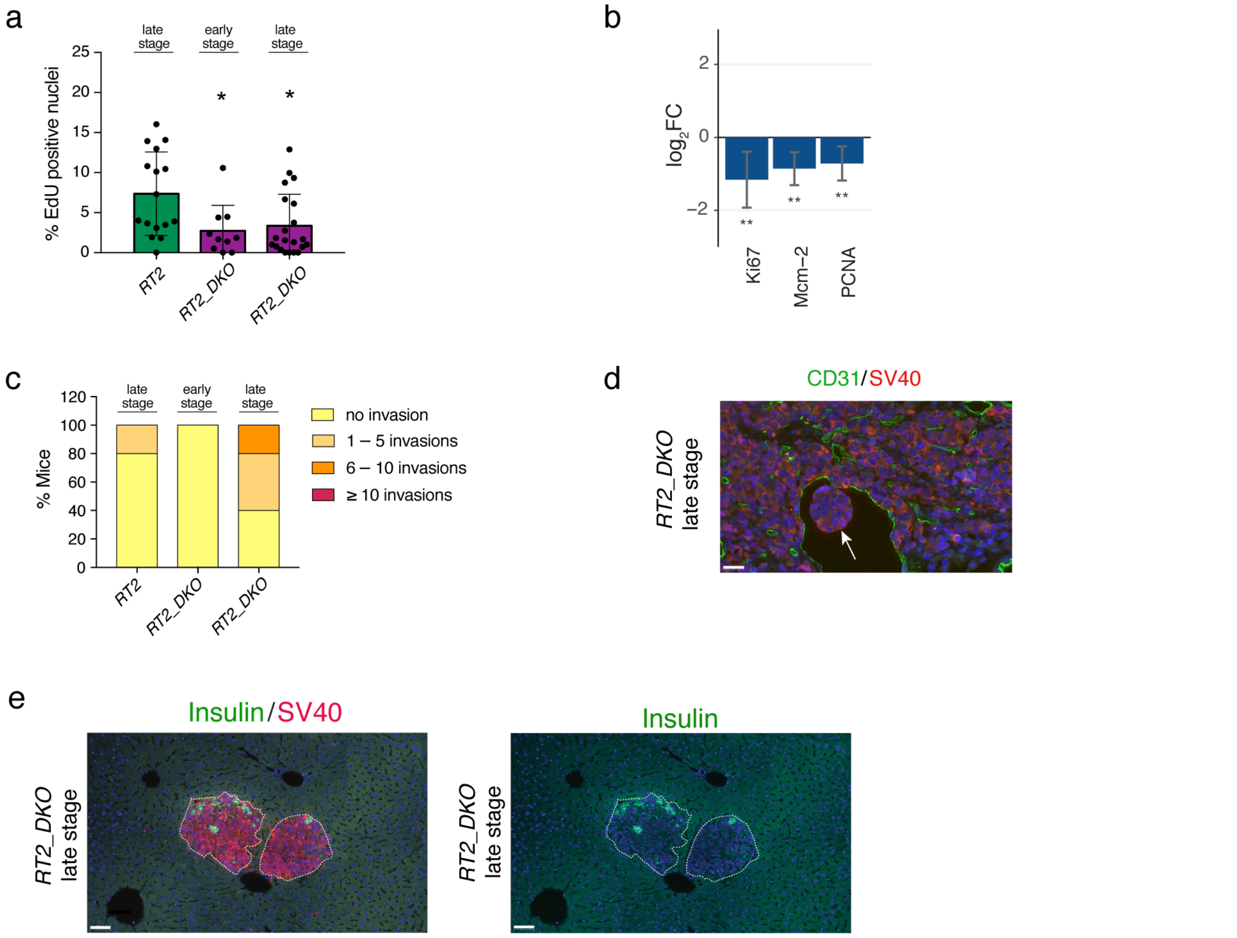

Supplementary Figure 1: Additional phenotypic characterization of *RT2_DKO* islets and tumors. **a** Percent of EdU-positive nuclei in lesions (n = 2 mice per group; late-stage *RT2*, early-stage *RT2_DKO*, late-stage *RT2_DKO*, n = 16,11,23 lesions, respectively). **b** Mean log2FC of proliferation genes in *RT2_DKO* vs. *RT2* islets of 6-week-old mice (RNA sequencing; *RT2*, *RT2_DKO*, n = 2,5, respectively). **c** Quantification of the number of vascular invasions identified in pancreatic sections, defined as the presence of SV40-positive cells in a CD31-positive vessel (n = 5 mice per group). **d** Representative SV40 and CD31 immunofluorescence staining of a vascular invasion in a late-stage *RT2_DKO* pancreas (scale bar = 20 µm). **e** Representative insulin and SV40 immunofluorescence staining of late-stage *RT2_DKO* liver (scale bar = 100 µm), showing no insulin expression in liver metastases. The small bright green regions are nonspecific autofluorescence of red blood cells. Error bars represent (**a**) SD or (**b**) 95% confidence intervals. Significance was assessed by (**a**) 1-way ANOVA with Dunnett's multiple comparisons test (vs. *RT2*) or (**b**) empirical Bayes method. * $P \leq 0.05$; ** $P \leq 0.01$; *** $P \leq 0.001$; **** $P \leq 0.0001$.

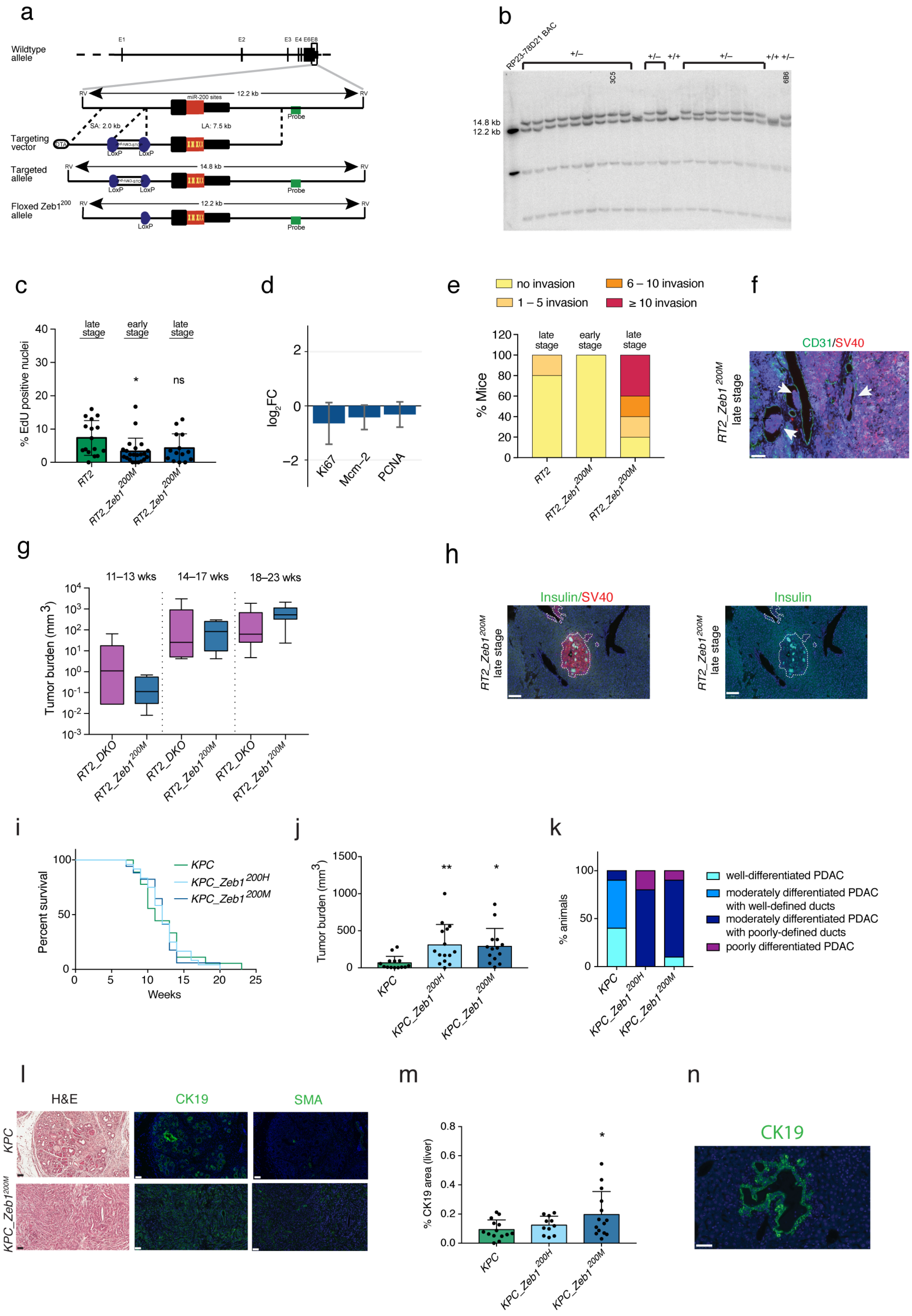
a
Wildtype allele
E1
E2
E3 E4 E6E8
RV
12.2 kb
miR-200 sites
Probe
SA: 2.0 kb
LA: 7.5 kb
Targeting vector
DTA
LoxP
LoxP
Targeted allele
14.8 kb
Floxed Zeb1200 allele
b
RP23-78D21 BAC
+/–
+/– +/+
+/–
+/+ +/–
3C5
6B6
14.8 kb
12.2 kb
c
% EdU positive nuclei
late stage
early stage
late stage
*
ns
RT2
RT2_Zeb1 200M
RT2_Zeb1 200M
d
log2FC
Ki67
Mcm-2
PCNA
e
no invasion
1 – 5 invasion
6 – 10 invasion
≥ 10 invasion
% Mice
late stage
early stage
late stage
RT2
RT2_Zeb1 200M
RT2_Zeb1 200M
f
CD31/SV40
RT2_Zeb1 200M late stage
g
Tumor burden (mm3)
11–13 wks
14–17 wks
18–23 wks
RT2_DKO
RT2_Zeb1 200M
h
Insulin/SV40
Insulin
RT2_Zeb1 200M late stage
i
Percent survival
Weeks
KPC
KPC_Zeb1 200H
KPC_Zeb1 200M
j
Tumor burden (mm3)
**
*
KPC
KPC_Zeb1 200H
KPC_Zeb1 200M
k
% animals
well-differentiated PDAC
moderately differentiated PDAC with well-defined ducts
moderately differentiated PDAC with poorly-defined ducts
poorly differentiated PDAC
KPC
KPC_Zeb1 200H
KPC_Zeb1 200M
l
H&E
CK19
SMA
KPC
KPC_Zeb1 200M
m
% CK19 area (liver)
*
KPC
KPC_Zeb1 200H
KPC_Zeb1 200M
n
CK19

Supplementary Figure 2: Generation and additional characterization of *RT2_Zeb1^{200}* mice.

a Replacement of the *Zeb1* 3'-UTR with a UTR that had point substitutions at each of the nine miR-200 sites. At the top is a schematic of the endogenous *Zeb1* locus (WT), zooming in on the region containing the *Zeb1* 3'-UTR. Below this is a schematic of the targeting vector, the successfully targeted allele, and the floxed *Zeb1*^{200} allele. Vertical bars labeled with E1–E8 indicate exons, with the shorter bar at the 3' end of E8 indicating the 3' UTR. The lengths of the short and long homology arms (SA and LA, respectively) are indicated. Location of probe for Southern blot, EcoRV sites (RV) and lengths of restriction fragments are also indicated for both alleles. Red vertical bars mark positions of nine miR-200 sites, and yellow X's indicate the disruption of these sites. A LoxP-flanked puromycin-resistance gene was used for positive selection and diphtheria toxin gene (DTA) was used for negative selection. **b** A Southern blot of genomic DNA digested with EcoRV and probed for a diagnostic fragment containing the *Zeb1* 3'-UTR (probe indicated in **a**). Lanes with DNA from wild-type V6.5s embryonic stem cells and successfully targeted clones are indicated (+/+ and +/–, respectively). A bacterial artificial chromosome containing the relevant fragment of the mouse genome (RP23-78D21 BAC) was also digested and included as a marker. After amplifying the knocked-in mutation with a primer external to the LA and sequencing the amplicon to confirm that the point mutations were introduced at the nine miR-200 sites, clones 3C5 and 6B6 were injected into C57BL/6j blastocysts to generate chimeras. **c** Percent of EdU-positive nuclei in lesions (n=2 mice per group; late-stage *RT2*, early-stage *RT2_Zeb1^{200M}*, late-stage *RT2_Zeb1^{200M}*, n=16,22,14 lesions, respectively). **d** Mean $\log_2$FC (with 95% confidence intervals) of proliferation genes in *RT2_Zeb1^{200M}* vs. *RT2* islets of 6-week-old mice (RNA sequencing; *RT2*, *RT2_Zeb1^{200M}*, n=2,4, respectively). **e** Quantification of vascular invasions identified in pancreatic sections, defined as the presence of SV40-positive cells in a CD31-positive vessel (n=5 mice per group). **f** Representative SV40 and CD31 staining of multiple vascular invasions in a late-stage *RT2_Zeb1^{200M}* pancreas (scale bar = 20 μm). **g** Box-and-whisker plots (box, 25th and 75th percentiles; central line, median) representing direct comparison of tumor burden

of *RT2_Zeb1*^{200M} vs. *RT2_DKO* mice (left to right: n=6,4,7,5,14,12 tumors). **h** Representative insulin and SV40 immunofluorescence staining of late-stage *RT2_Zeb1*^{200M} liver (scale bar = 100 μm), showing no insulin expression in liver metastases. The small bright green regions are nonspecific autofluorescence of red blood cells. **i** Percent survival of *KPC*, *KPC_Zeb1*^{200H}, and *KPC_Zeb1*^{200M} mice (n=18,24,17). **j** End-stage tumor burden of *KPC*, *KPC_Zeb1*^{200H}, and *KPC_Zeb1*^{200M} mice (n=14,15,14). **k** Grade of the highest grade tumor in *KPC*, *KPC_Zeb1*^{200H}, and *KPC_Zeb1*^{200M} (n=10) mice. **l** Representative H&E, Ck19, and SMA immunofluorescence stainings of moderately differentiated PDAC of *KPC* and *KPC_Zeb1*^{200M} mice (scale bar = 50 μm). Note that ducts are better defined in *KPC* than *KPC_Zeb1*^{200M}. **m** Quantification of CK19 immunofluorescence staining in end-stage livers of *KPC*, *KPC_Zeb1*^{200H}, and *KPC_Zeb1*^{200M} mice (n=14,11,14). **n** Representative Ck19 staining of *KPC_Zeb1*^{200M} liver metastasis (scale bar = 50 μm). **c, j, m** Data are plotted as mean ± or + SD. Significance was assessed by (**c, g, j, m**) 1-way ANOVA with Dunnett's or Tukey's multiple comparisons test (vs. *RT2*), (**d**) empirical Bayes method, or (**i**) Mantel Cox test. * P ≤ 0.05; ** P ≤ 0.01; *** P ≤ 0.001; **** P ≤ 0.0001.

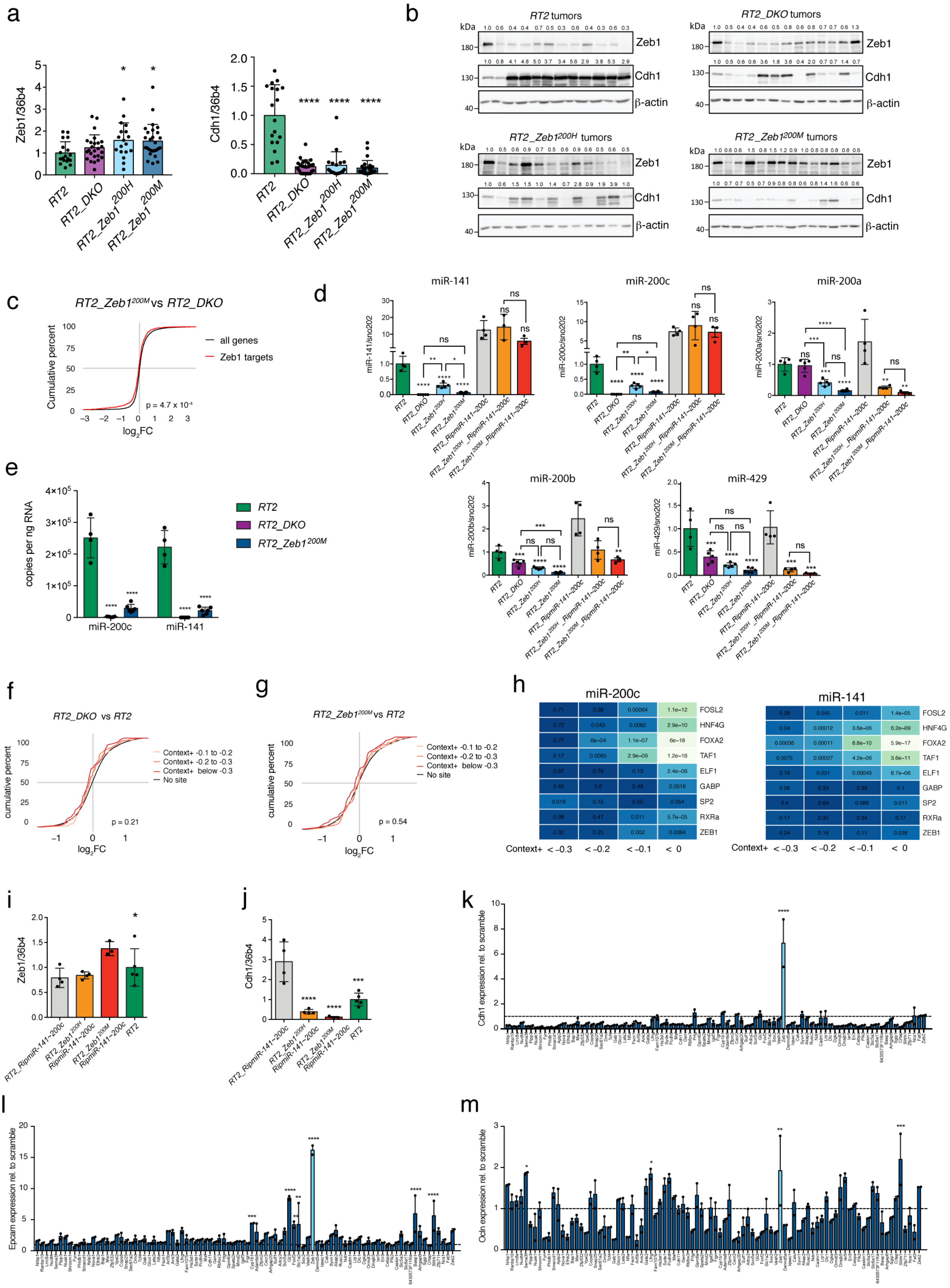
a
Zeb1/36b4
Cdh1/36b4
RT2
RT2_DKO
RT2_Zeb1 200H
RT2_Zeb1 200M
b
RT2 tumors
RT2_DKO tumors
RT2_Zeb1200H tumors
RT2_Zeb1200M tumors
kDa
Zeb1
Cdh1
β-actin
c
RT2_Zeb1200M vs RT2_DKO
Cumulative percent
all genes
Zeb1 targets
p = 4.7 x 10-4
log2FC
d
miR-141
miR-200c
miR-200a
miR-200b
miR-429
miR-141/sno202
miR-200c/sno202
miR-200a/sno202
miR-200b/sno202
miR-429/sno202
RT2_RipmiR-141~200c
RT2_Zeb1200H _RipmiR-141~200c
RT2_Zeb1200M _RipmiR-141~200c
ns
e
copies per ng RNA
RT2
RT2_DKO
RT2_Zeb1200M
miR-200c
miR-141
f
RT2_DKO vs RT2
cumulative percent
Context+ -0.1 to -0.2
Context+ -0.2 to -0.3
Context+ below -0.3
No site
p = 0.21
log2FC
g
RT2_Zeb1200M vs RT2
p = 0.54
h
miR-200c
miR-141
FOSL2
HNF4G
FOXA2
TAF1
ELF1
GABP
SP2
RXRa
ZEB1
Context+ < -0.3 < -0.2 < -0.1 < 0
i
Zeb1/36b4
j
Cdh1/36b4
k
Cdh1 expression rel. to scramble
l
Epcam expression rel. to scramble
m
Ocln expression rel. to scramble

Supplementary Figure 3: Additional analysis of Zeb1 and miR-200 regulation in *RT2_DKO* and *RT2_Zeb1^{200}* mice.

a Relative expression of *Zeb1* and *Cdh1* in end-stage tumors of *RT2, RT2_DKO, RT2_Zeb1^{200H}*, *RT2_Zeb1^{200M}* mice (n=18,25,25,27, respectively), measured by qPCR (vs. 36b4). **b** Immunoblots showing ZEB1 and CDH1 expression in end-stage tumors (*RT2*, *RT2_DKO*, *RT2_Zeb1^{200H}*, *RT2_Zeb1^{200M}*, n=10,13,10,12). Each blot was loaded with the same first two *RT2_DKO* tumors to enable comparison, with the first sample as calibrator. Quantification is normalized to beta-actin. **c** Cumulative distributions of $\log_2$FC of Zeb1 targets in islets. **d** Relative expression (Taqman qPCR, vs. sno202) of miR-200 in islets (*RT2, RT2_DKO*, *RT2_Zeb1^{200H}*, *RT2_Zeb1^{200M}*, *RT2_RipmiR-141~200c*, *RT2_Zeb1^{200H}_RipmiR-141~200c*, *RT2_Zeb1^{200M}_RipmiR-141~200c*, n=4,5,5,6,4,4,3, respectively). Data were analyzed in two groups, vs. *RT2* or *RT2 RipmiR-141~200c*. **e** Absolute quantification of miR-200c and miR-141 in islets (*RT2*, *RT2_DKO*, *RT2_Zeb1^{200M}*, n=4,5,6, respectively) by Taqman qPCR. **f, g** Cumulative distributions of $\log_2$FC of miR-141 predicted targets in islets of (**f**) *RT2_DKO* vs. *RT2* and (**g**) *RT2_Zeb1^{200M}* vs. *RT2*. P-values shown are for context+ scores < –0.3. **h** Overlap between miR-200 predicted targets and ChIP data sets of transcription factors expressed in HepG2 cells with indicated Context+ score cutoffs. P-values represent the probability of a given miR-200 target also being in a given ChIP data set. **i, j** Relative expression of (**i**) Zeb1 and (**j**) Cdh1 in islets of *RT2_RipmiR-141~200c, RT2_Zeb1^{200H}_RipmiR-141~200c, RT2_Zeb1^{200M}_RipmiR-141~200c*, and *RT2* mice (n=4,4,3,5 respectively). Statistics are vs. *RT2_RipmiR-141~200c*. **k–m** Relative expression of (**k**) *Cdh1*, (**l**) *Epcam,* and (**m**) *Ocln* following siRNA knock-down of differentially expressed miR-200c predicted targets in a *RT2_DKO* cell line (vs. scrambled control). N=2 technical replicates. **c, f, g** RNA sequencing of islets from 6-week-old mice (*RT2*, *RT2_DKO*, *RT2_Zeb1^{200M}*, n=2,5,4 mice, respectively). **a, d, e, i, j** Data are plotted as mean ± SD or (**k–m**) SEM. Significance was assessed by (**a, d, e, i–m**) 1-way ANOVA followed by Dunnett's multiple comparisons test, (**h**) hypergeometric test, or (**c, f, g**) competitive gene set test. * $P \leq 0.05$; ** $P \leq 0.01$; *** $P \leq 0.001$; **** $P \leq 0.0001$.

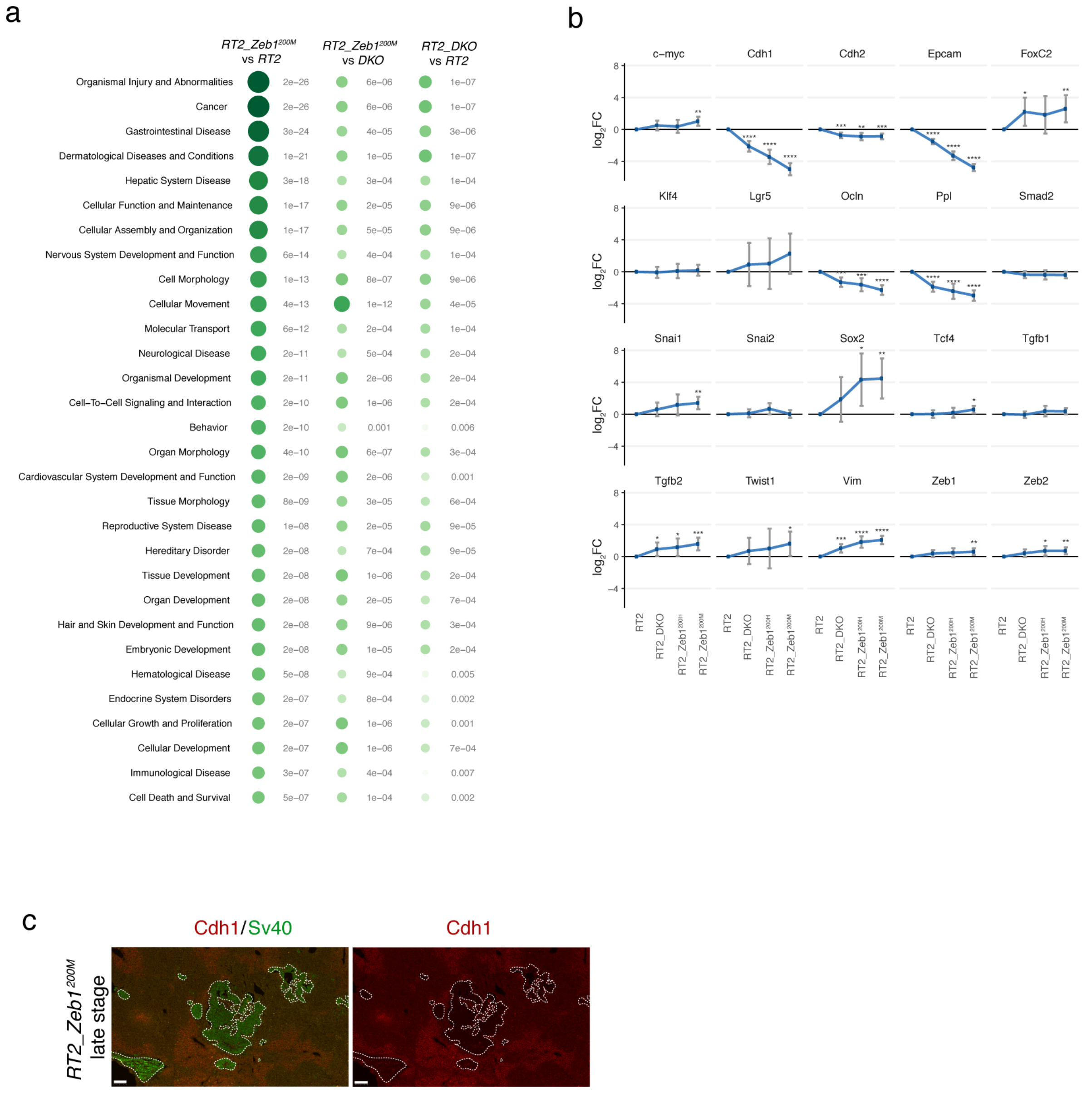

Supplementary Figure 4: EMT gene regulation in islets of *RT2_DKO* and *RT2_Zeb1[200M]* mice. **a** Ingenuity biological function analysis of differentially expressed genes in *RT2_Zeb1[200M]* vs. *RT2*, *RT2_DKO* vs. *RT2*, and *RT2_Zeb1[200M]* vs. *RT2_DKO* islets of 6-week-old mice. Categories shown were selected based on having a p-value < 10^{-6} in at least one comparison. **b** Log_2FC of select EMT and stemness genes in islets. Error bars represent 95% confidence intervals. **c** Representative CDH1 and SV40 immunofluorescence staining of a late-stage *RT2_Zeb1[200M]* liver (scale bar = 100 µm), with metastases outlined. **a, b** RNA sequencing of islets of 6-week-old mice (*RT2, RT2_DKO, RT2_Zeb1[200H], RT2_Zeb1[200M]*, n = 2,5,3,4, respectively). Signficance was assessed by (**b**) empirical Bayes method. * P ≤ 0.05; ** P ≤ 0.01; *** P ≤ 0.001; **** P ≤ 0.0001.

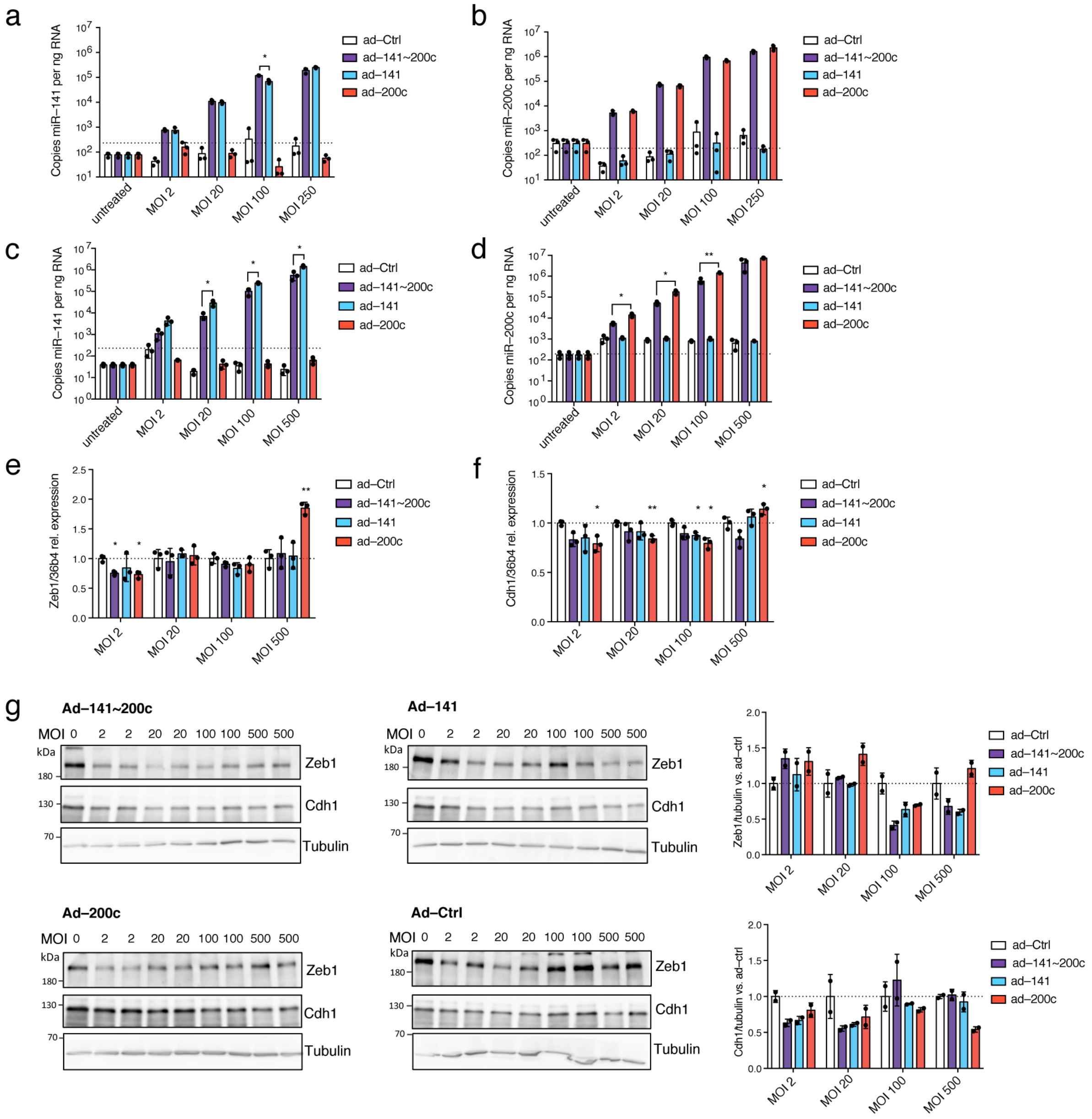

Supplementary Figure 5: Additional analysis of the distinct functions of miR-200c and miR-141 in EMT in *RT2_DKO* and *RT2_Zeb1^{200M}* cells. **a–d** Absolute quantification of miR-141 and miR-200c in (**a, b**) *RT2_DK0*-derived cells or (**c, d**) *RT2_Zeb1^{200M}*-derived cells infected with ad-141, -200c, -141~200c, or ad-Ctrl (n = 3 replicates). **e, f** *Zeb1* and *Cdh1* expression in *RT2_Zeb1^{200M}*-derived cells infected with ad-141, -200c, -141~200c, or ad-Ctrl, measured by qPCR (n = 3 replicates). Expression was normalized to Ad-Ctrl at the corresponding MOI. **g** Immunoblots showing ZEB1 and CDH1 expression in lysates of *RT2_Zeb1^{200M}*-derived cells infected with the indicated adenoviruses at the range of MOIs shown. Zeb1 and Cdh1 band densities were quantified and normalized to tubulin and to the appropriate ad-Ctrl (right). **a–g** Data are plotted as mean ± SD. **a–f** Significance was assessed by multiple two-tailed t-tests (at each MOI) with Holm-Sidak correction for multiple comparisons.
* P ≤ 0.05; ** P ≤ 0.01; *** P ≤ 0.001; **** P ≤ 0.0001.

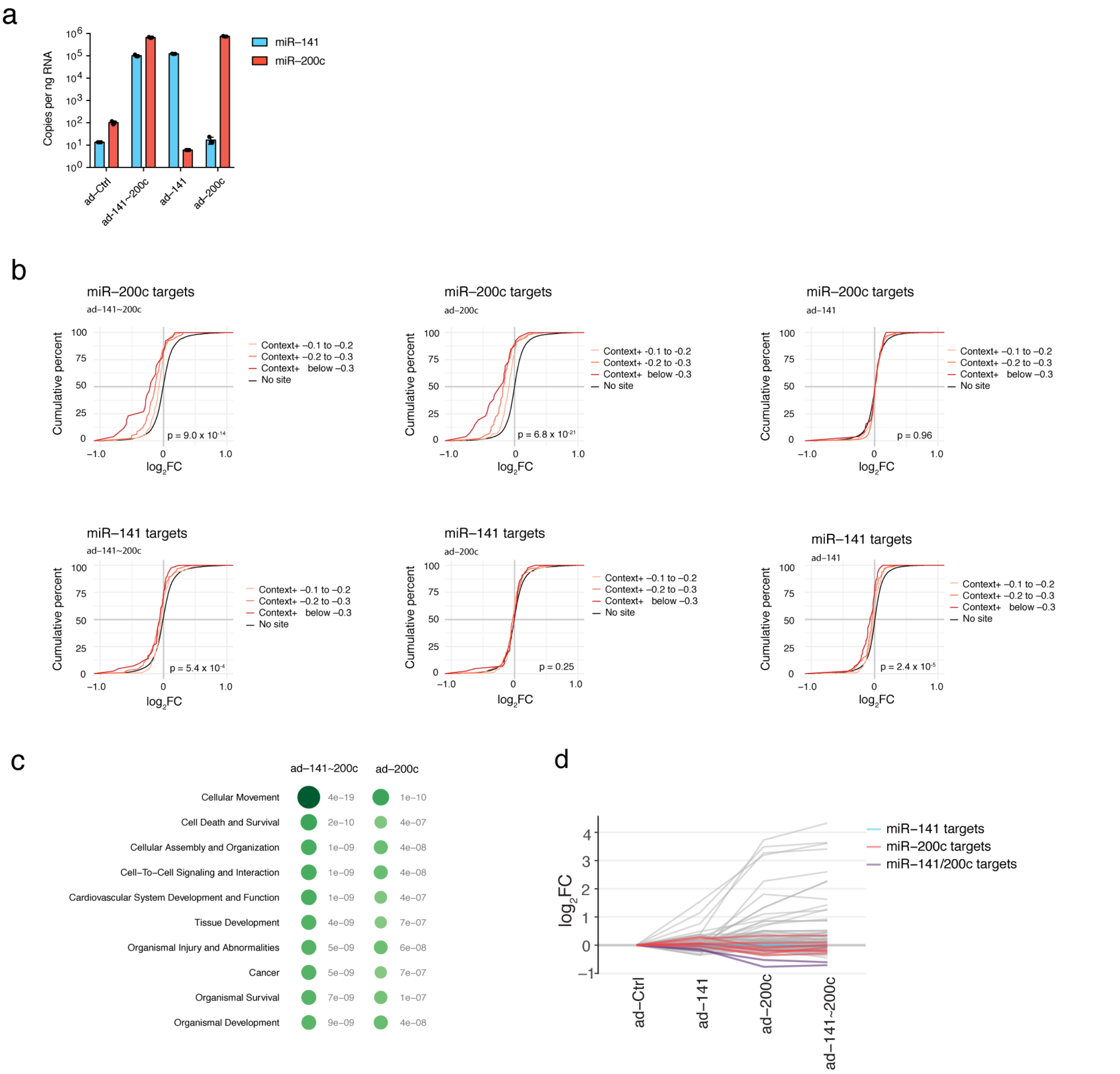

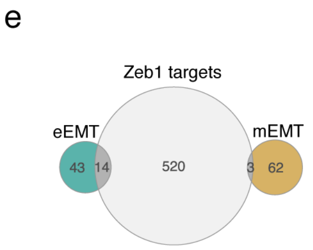

Supplementary Figure 6: Additional analysis of the distinct functions of miR-200c and miR-141 in EMT by RNA sequencing.
a Absolute quantification of miR-200 in *RT2_DKO* cells infected with ad-Ctrl, ad-141, ad-200c, or ad-141~200c (MOI 100) and used for RNA sequencing (n = 2). **b** Cumulative distributions of $\log_2$FC showing regulation of miR-200c or miR-141 predicted targets in ad-141~200c-, ad-141-, or ad-200c-infected *RT2_DKO* cells of RNA sequencing experiment (n = 2). **c** Top 10 most regulated biological functions (Ingenuity) in ad-141~200c vs. ad-Ctrl and ad-200c vs. ad-Ctrl treatment of *RT2_DKO* cells. There were not a sufficient number of significantly regulated genes in ad-141 vs. ad-Ctrl to perform similar analysis. **d** Overlap between predicted miR-141, miR-200c, and miR-141~200c targets and EMT genes regulated in adenovirus-infected *RT2_DKO* cells (Figure 5f). **e** Venn diagram depicting the overlap between Zeb1 ChIP targets, eEMT genes, and mEMT genes. **a** Data are depicted as mean ± SD. Significance was assessed by (**b**) competitive gene set test. **b–d** RNA sequencing of adenovirus-infected *RT2_DKO* cells (n = 2 technical replicates for each adenoviral infection). * $P \leq 0.05$; ** $P \leq 0.01$; *** $P \leq 0.001$; **** $P \leq 0.0001$.

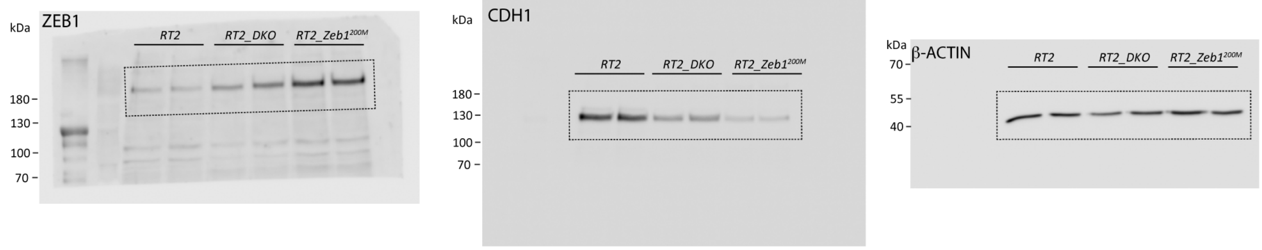

Supplementary Figure 7: Uncropped immunoblots of islets from 6-week-old mice.
Full scans of immunoblots situated in Figure 3b, with dotted line indicating rough region of cropping.

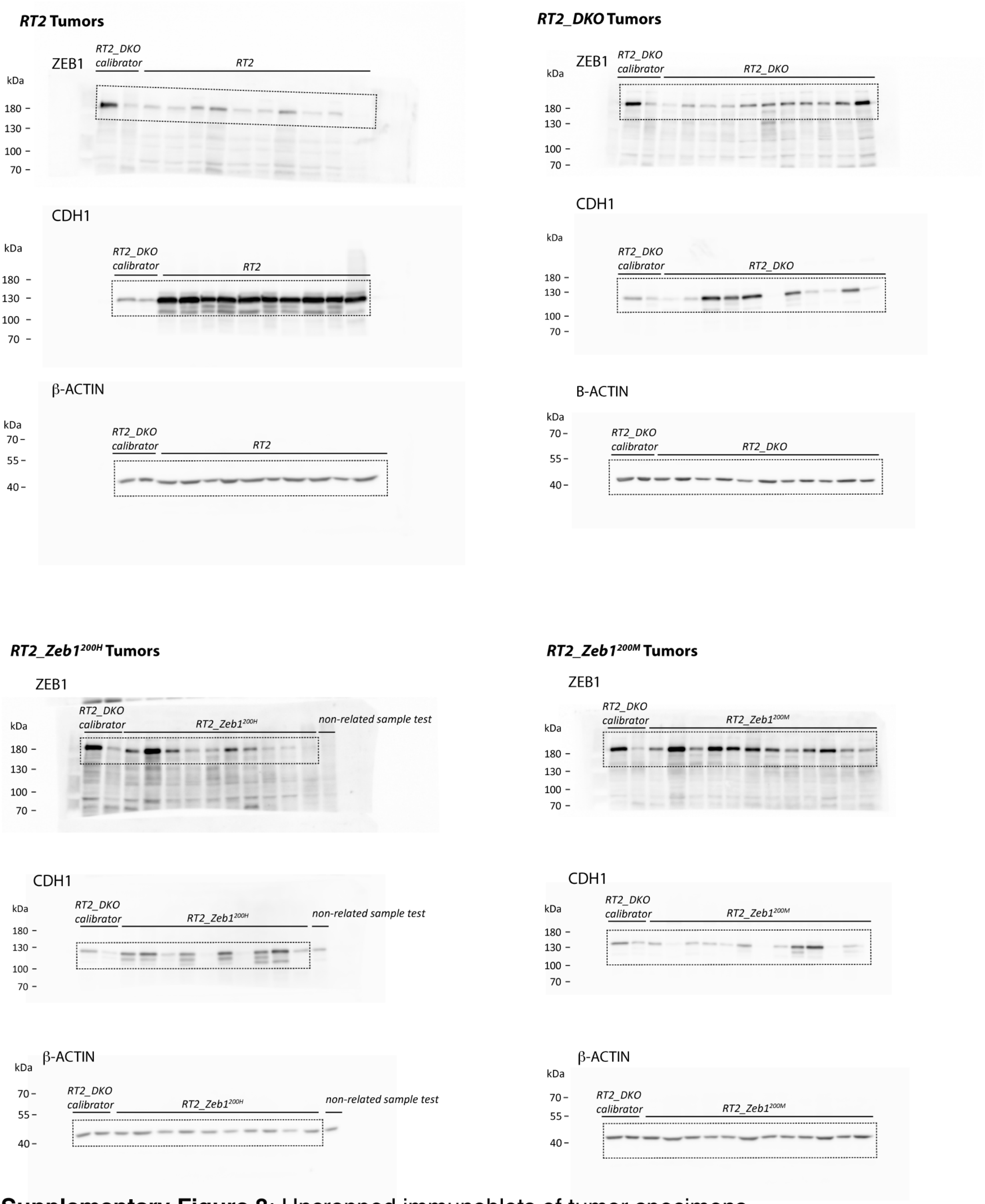

Supplementary Figure 8: Uncropped immunoblots of tumor specimens.
Full scans of immunoblots situated in Supplementary Figure 3b, with dotted line indicating rough region of cropping.

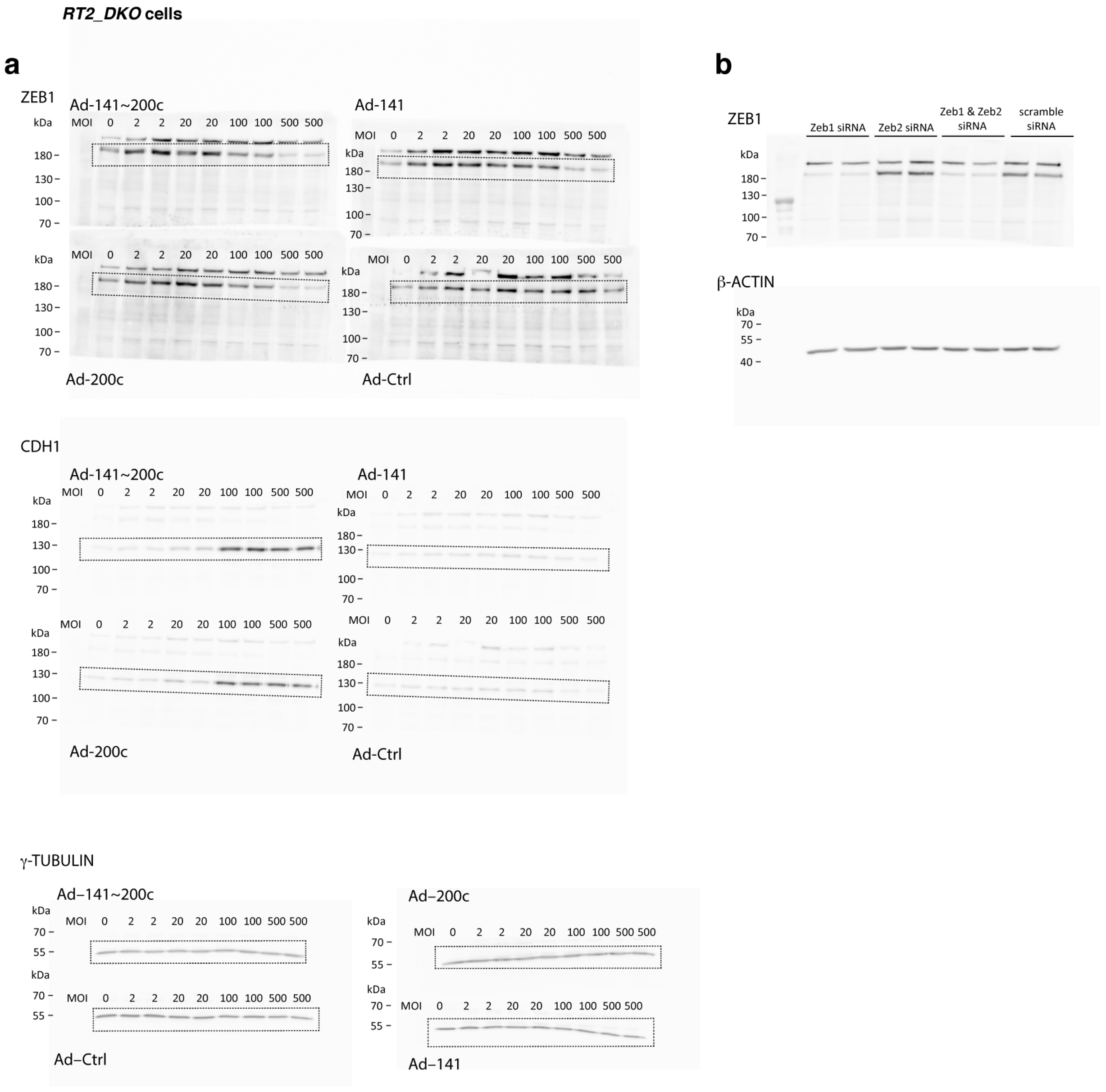

Supplementary Figure 9. Uncropped immunoblots of *RT2_DKO* cells treated with ad-200.
a Full scans of immunoblots situated in Figure 5c, with dotted line indicating rough region of cropping.
b Zeb1 siRNA knockdown in *RT2_DKO* cells indicating the correct band.

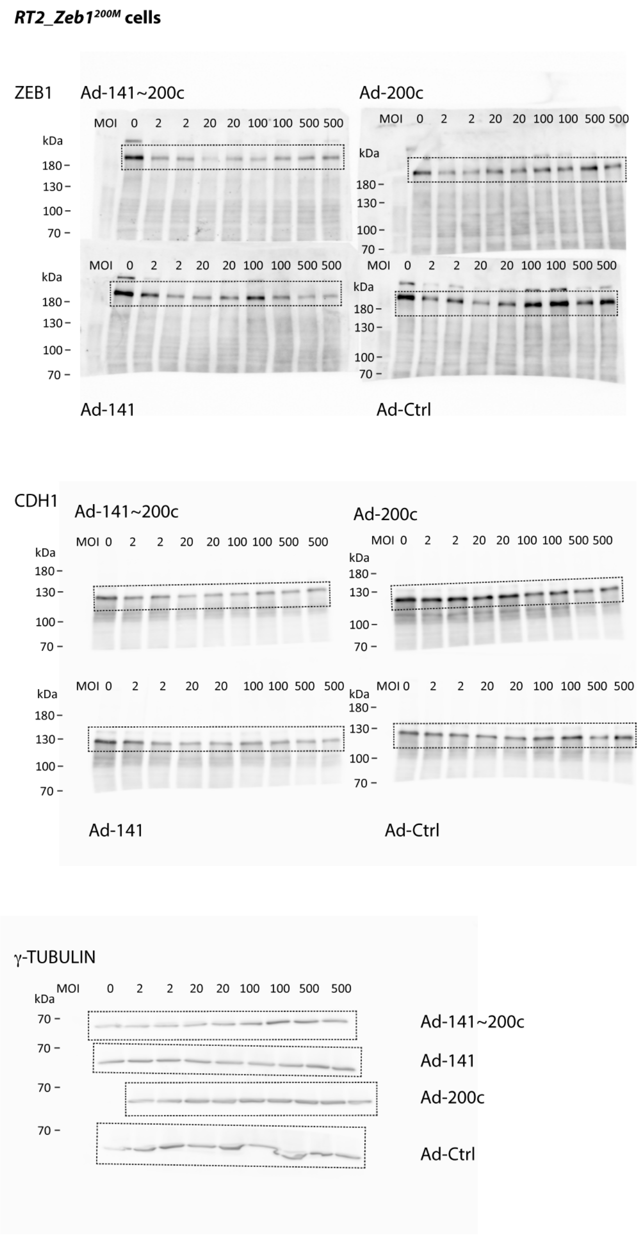

Supplementary Figure 10: Uncropped immunoblots of *RT2 Zeb1*^{200M} cells treated with ad-200.
Full scans of immunoblots situated in Figure S5g, with dotted line indicating rough region of cropping.

Supplementary Table 1

Primers, Taqman Assays, and siRNAs

Adenovirus generation	Primers
pre-mir-141 and ~200bp flanking region	5'-AAGCCCCTTCTTCCGAGTTA-3' and 5'-GTTCCCAGGGTGAAAAGACA-3'
pre-mir-200c and ~200bp flanking region	5'-GGCTCGCAGGTGGATAGTAG-3' and 5'-TAGACAATCCCAAGGCCAAG-3'

Zeb1 3'UTR amplification	Primers
WT 3'UTR and Zeb1^{200M} 3' UTR	5'-GCTCACTACTGTGTAATG-3' and 5'-GACAATTTTCCAAAGGAA-3'

Gene	Species	qPCR Primers
Cdh1	Mouse	5'-ACTGTGAAGGGACGGTCAAC-3' and 5'-GGAGCAGCAGGATCAGAATC-3'
Epcam	Mouse	5'-GCGGCTCAGAGAGACTGTG-3' and 5'-CCAAGCATTTAGACGCCAGTTT-3'
Ocln	Mouse	5'-TTGAAAGTCCACCTCCTTACAGA-3' and 5'-CCGGATAAAAAGAGTACGCTGG-3'
Zeb1	Mouse	5'-GCCAGCAGACCAGACAGTATT-3' and 5'-TCACACTCGTTGTCTTTCACG-3'
36b4	Mouse	5'- GCCGTGATGCCCAGGGAAGACA -3' and 5'-CATCTGCTTGGAGCCCACGTTG-3'
INS	Human	5'-GAGGCCATCAAGCAGATCACT-3' and 5'-ATTGTTCCACAATGCCACGC-3'
MAFA	Human	5'-TTGAGCGGAGAACGGTGATT-3' and 5'-GAAGGTGGGAACGGAGAACC-3'
PDX1	Human	5'-TGCCTTTCCCATGGATGAAGT-3' and 5'-GCGGCCGTGAGATGTACTTG-3'
KIR6.2	Human	5'-CCCAGGTGGAGGTAAGGAAGA-3' and 5'-GTGTCAGCACGTATTCCTCG-3'
NKX6.1	Human	5'-AGGGCTCGTTTGGCCTATTC-3' and 5'-AGAGGCTTATTGTAGTCGTCGT-3'
SUR1	Human	5'-CCGCACGTCTTCCTACTCTT-3' and 5'-GATTCGGTCACCCCATCAGA-3'
ZEB1	Human	5'-TTACACCTTTGCATACAGAACCC-3' and 5'-TTTACGATTACACCCAGACTGC-3'
ZEB2	Human	5'-CAAGAGGCGCAAACAAGCC-3' and 5'-GGTTGGCAATACCGTCATCC-3'
SNAI2	Human	5'-CGAACTGGACACACATACAGTG-3' and 5'-CTGAGGATCTCTGGTTGTGGT-3'
DLC1	Human	5'-CCAAACCCAAGACTACGGCTA-3' and 5'-GCTGTGCATACTGGGGGAA-3'
CDH1	Human	5'-CGAGAGCTACACGTTCACGG-3' and 5'-GGGTGTCGAGGGAAAAATAGG-3'
EPCAM	Human	5'-ATAACCTGCTCTGAGCGAGTG-3' and 5'-TGCAGTCCGCAAACTTTTACTA-3'
DSP	Human	5'-GCAGGATGTACTATTCTCGGC-3' and 5'-CCTGGATGGTGTTCTGGTTCT-3'
OCLN	Human	5'-ACAAGCGGTTTTATCCAGAGTC-3' and 5'-GTCATCCACAGGCGAAGTTAAT-3'
TWIST1	Human	5'-GTCCGCAGTCTTACGAGGAG-3' and 5'-GCTTGAGGGTCTGAATCTTGCT-3'
VIM	Human	5'-AGTCCACTGAGTACCGGAGAC-3' and 5'-CATTTCACGCATCTGGCGTTC-3'

Taqman Assay name	assay ID
hsa-miR-200a	000502
hsa-miR-200b	002251
hsa-miR-200c	002300
hsa-miR-141	000463
mmu-miR-429	001077
snoRNA202	001232
hsa-miR-16	000391

Gene	siRNA sequence (pools of 4)
Scramble control	#D-001810-10, Dharmacon
6430573F11Rik	GAUUAAACGACACGGCUCA
6430573F11Rik	CCAAAGCCUUCAUGCGUUA

6430573F11Rik	GUAGAGAUUGCCCGGAAUA
6430573F11Rik	ACAGAAGAACCGACGCUUU
Adamts8	UGUCAAUGGCAGCCCGAAU
Adamts8	CAAAUAUGUCUCCGAACUA
Adamts8	CGUGAAUGUGAUAAUCCAA
Adamts8	AGAAGUCGGGAUAGGUUAA
Adcy2	GCCAAUGGGUCAACAUAAU
Adcy2	GAAAUCAUCAGGCAUCAUA
Adcy2	UAUCAACCAUGGACCUGUA
Adcy2	GAACUUGGCGGGAGCUUAC
Akap2	GAAAGUACAAGGAGCGCAA
Akap2	GAUGGAACUUACAACGGAA
Akap2	CGGACAAGCCACCGACCAU
Akap2	CUGGAGGCCACACGGGUUA
Ank3	GGCUAUAAGGUGAAGACGA
Ank3	CCUCAUACCUCACGGGAGA
Ank3	CCUUGUGAGCGGACGGAUA
Ank3	GGACUACGGCUGACGGCAA
Aplp2	GAAUGACCGCCGACGCAUA
Aplp2	AGGAGGAGGUUCUUCAGUA
Aplp2	GAACAAAGAUCGCCUGCAU
Aplp2	GUACAAAGUUCCUUAUGUU
Arhgap20	GCGAUGAGUUUGUAUAAUA
Arhgap20	GGGCAGUACCCUUGUGAUA
Arhgap20	GUGGGUAAAUUCUGGAAAA
Arhgap20	AGAGAAAACUAGUGGGUAA
Arhgap6	GGAUAUAUAUGACGUGUUA
Arhgap6	GAGUCUACAUCACCGAAUA
Arhgap6	GAGUAAGACAAUUGCGUGA
Arhgap6	GCUAAUGACCGGGCAUAUA
Arl5b	ACGCCUGGCUAUUACGAAA
Arl5b	GGACGAGCAUAACGUGUCA
Arl5b	CACAAAUACUUGCGUUUAA
Arl5b	GGGAUAAAGUGUGUAUGUA
Bap1	GGGUCAUCAUGGAGCGAAU
Bap1	CCAUCAGAUACAAGCGGAA
Bap1	GUGUGUAGAAGCCGAGAUU
Bap1	CCAUAGGGCUUGGUAGUAA
Basp1	GCUACAAUGUGAACGACGA
Basp1	GCAAAGCGUGGCCGUCAAA
Basp1	GAGGCAAGCUGAGCAAGAA
Basp1	CCACCGAGGUCAAGGAGAG
Cadm1	CGUAACUUGAUGAUCGAUA
Cadm1	AGCGAAGACUAGAAACGUA
Cadm1	CGUGUGAAGCCAUCGGGAA
Cadm1	GGAAAGUGGCAUAUAGGGA
Calu	GGGAGGCCUUAGUACGACA

Calu	CGAGAUAAGAACCGGGAUG
Calu	GCCUCAGCUCAGCGAUAAA
Calu	CCAAGGAGGAGAUUGUCGA
Caskin1	GGAAGAGACUAGCGCGUGU
Caskin1	CCACAAGUGUCGGGCAUAA
Caskin1	CCAUUGGGCCUGACGGUGA
Caskin1	CAGUGAAGGCGGAGGACGU
Cdh11	GCUUAUAGCUUGAAGAUAG
Cdh11	AGAUAACACUGCAGGAGUA
Cdh11	GGUCAUCGUUGUGCUGUUU
Cdh11	CAAUUGAUCGUCAUACUGA
Cebpa	GAGCCGAGAUAAAGCCAAA
Cebpa	CCUGAGAGCUCCUUGGUCA
Cebpa	GGAGUUGACCAGUGACAAU
Cebpa	CUAUAGACAUCAGCGCCUA
Cecr2	GUUCAGAAAUUGCCGAAAA
Cecr2	GAAGAGAUAUCACGCCUCA
Cecr2	GGAGGUUUAUAUUGUAACA
Cecr2	GAGAUGACGUGGAGUUUAU
Cops8	GGUCAGUUGGACAGCGAAU
Cops8	AGUAUUACACAUCGAGUUU
Cops8	ACAUAGAAGAUGAAGCGCA
Cops8	UGUACUUGCUCCAGAACGA
Crtap	CUAUUUACAGUUUGCGUAU
Crtap	CUUCAUGGUUACACGUCUU
Crtap	GGGGAGAACUGGAGAACGU
Crtap	AGAAAUUUGUGGCGACCAU
Cyp1b1	CCAUUAAGCCCAAGUCGUU
Cyp1b1	GCGACAGUGGCAACGGAAA
Cyp1b1	ACAUUAAGCAGAUAGAUCA
Cyp1b1	CGAAUAGAUUACAGGCUUA
Dennd5a	GAGGAUGGUUCUCGGACAU
Dennd5a	CCUCAUGGCCUUUGGCGUU
Dennd5a	CUGACAAGAGGAACGGGAA
Dennd5a	ACACAGAACACUUGCGCUU
Dgka	GGACAAGAGUGCAAACACA
Dgka	GAAUAGAGGGUGCAAUUGA
Dgka	GCGAGUGGCCGAAUAUCUA
Dgka	GUUAUGAAGGUGAGAAUUU
Dlc1	GGAUCAAGGUUCCAGACUA
Dlc1	CGCCUGAGCAUCUAUGAUA
Dlc1	AGAUGAGCCUUGCGCCAUA
Dlc1	GUGCAGAGCUGAUUUAAGG
Dmd	AGUAACAGUUCACGGUAAA
Dmd	GGAGGAGAGACUCGGGAAA
Dmd	GGGAACAAAUCAUGGUAAA
Dmd	CGUAAGAUGAGAAAGGAAA

Dnajb5	GCUCCAACCCCUUCGAUAU
Dnajb5	GAGUAGGGGUCCAAGGCGA
Dnajb5	CAACAUCCCAGCCGACAUU
Dnajb5	GUUGUAUAGAAGUCGAAUA
Efnb2	CCAAAUUUCUACCCGGACA
Efnb2	GCAUAUUUGUGCCGUAAUU
Efnb2	CCGCGGAAGGAAAGGCGAA
Efnb2	GUUCAGAUCUUACCGUCUA
Eri3	GUUCAAUGCUAGCGCCGUU
Eri3	GUCGAUGAGUGGAUGGCGA
Eri3	GUACAGAGCUCACCGGGAU
Eri3	UCUUAGAUCCAAACGUCAA
Fam107b	GCAAGAGGGUUGUACUACU
Fam107b	GCAUAGAAUAGCAGAUGUA
Fam107b	CCAUUUGAGUGUACAUAAU
Fam107b	GUGAUGAGGCCUUUAGAGA
Fat3	CGACAGCUAUGUAGAGCGU
Fat3	UCUUGGAGCUCAAUCGCAA
Fat3	GGAACAGUUACAUCAAGUA
Fat3	GCAUGAUUCUGAAGAUUGU
Fign	ACUCGUGAAUGGUCGGAAA
Fign	GGUUCAAGCUAGACGACAC
Fign	ACUAAAGAGAUGAACGGUU
Fign	AGUUAAGGCCCGUUACAUA
Foxf2	CAUCAUCACCAGAGCGUGU
Foxf2	GGAGUUCUGCUCACCGAUA
Foxf2	ACACUAGCCCUGCGGCACA
Foxf2	UAAUAUCCCAGAAGAGCGA
Fscn1	CCGACGAGAUCGCGGUAGA
Fscn1	GCGUUACCCGCAAGCGCUA
Fscn1	GGAGAUCGACCGCGACACA
Fscn1	GGUAGUGAUUCCUCGGUCA
Fyn	GGUGCGAAGUUUCCCAUUA
Fyn	GCACGUGACUCGUUGUUUC
Fyn	UAACUGUGGUUUCAUCAAG
Fyn	CCACGUCAAACAUUAUAAA
Gab1	GAGACAAACAAGUCGAAUA
Gab1	CAAGAAGCCUAUUCGGAUU
Gab1	CGAUUCACCUUUCGCUAUA
Gab1	CAUCAAGCACGGAGGCCGA
Gata2	CGCCGCAGCUCAUGACUAU
Gata2	GCACAAUGUUAACAGGCCA
Gata2	GAAGAGCGGGCACCUGUUG
Gata2	CGACAACCACCACCUUAUG
Gem	GAAGAUACAUAUGAGCGUA
Gem	CAACCAAGUGCACGGGCAA
Gem	UUACUUAUGUGUCGUAGAA

Gem	UUGAUAACCUGCAGCGCAA
Glcci1	UGGGAGUAGCUCACCGUUA
Glcci1	AAGCAAAGUAGUCGGCAUA
Glcci1	CAGCUUUCCUCCCGGGUUU
Glcci1	GCAUUGAUCCCUAGGAGAU
Gli3	GAACAACCCUAGUCAAGGA
Gli3	GCUCUACCGUGCAGAAUUA
Gli3	GCUUAGUGCUGCAAAAUUA
Gli3	CCUCACAUAUCAACAUCUA
Gpm6a	GUAUAAACCUAGGUAGUAU
Gpm6a	UAACAUUGUCCAACGCAAA
Gpm6a	GGGUAUUACUAGCGUUUCU
Gpm6a	CUUCAAAGGGACCGUUGUA
Hs3st1	AGAUCAACGCCUCGAACUU
Hs3st1	CUUGUUAAAUGGAGGGCGU
Hs3st1	GGAGAAGACACCCGCCUAU
Hs3st1	UGAUAAACUGCACGAAUAC
Ier5	GGUCUAGCUUCUCGGGACU
Ier5	UGGAAGAGGACGAUCGAUC
Ier5	GAGAGGGACUUUACACACA
Ier5	GAGUUCAAGCUUGGAAAUA
Igsf3	GGAGGAGGACAUAUCGCAA
Igsf3	AGAACAUUCCCGACCGGUA
Igsf3	AAAUACAACUGCCGGGUGA
Igsf3	GCAGAAGGGUGGCGAGAAC
Iqsec1	UCACUAAUGACCCGAGUUA
Iqsec1	UCAAUAUGUUAUCGGAUCA
Iqsec1	GCGGAGGCCCUUUCAGAUU
Iqsec1	GAUAAUGAGAGCUUGUGAU
Lats2	GAAAUAGCCGGCAGCGAUU
Lats2	GCGGCAAUAUUUAGACUUU
Lats2	GGAUUCAGGUGGACUCACA
Lats2	GGAUGGAGGUCUUCCCUGA
Lfng	CUGUGGAGUAUGACCGAUU
Lfng	CCACAGAACGGAUCAGCGA
Lfng	GUGCAUAGCCUCUCCGAGU
Lfng	CACAAGGAGAUGACGUUCA
Lrp1	GCUGUAACAUGUUCGAUGA
Lrp1	GACCAGUGUUCUCUGAAUA
Lrp1	GGAGUCACUUACAUCAAUA
Lrp1	GCAUUGGUGUUCAGCUUAA
Lrrc8a	CAAGUAUGACCUAGAUCGA
Lrrc8a	UGACCAACCUGACGCAGAU
Lrrc8a	GCCUCAAGGUGCUGCGACU
Lrrc8a	GAUUACAUCUCCAUCGUCA
Maf	CCUACAAGGAGAAAUACGA
Maf	GGCAAAUAUUAUAGAACGU

Maf	AGCAACGGCUUCCGAGAAA
Maf	UUGAAGACUUAGCGCAUGA
Mmd2	CCAAAUAUGCAAGGUUUAU
Mmd2	CAUGAACGGUACAAGCUUG
Mmd2	GCUAUGUGGUGAUGGGUUU
Mmd2	GACCAUAUCUGCCUGGAUC
Msn	CGGAUUAACAAGCGGAUCU
Msn	UUUCAGUAUUAGCGACUUA
Msn	AGUUGGAAAUGGCUCGAAA
Msn	GAGAAGAUUGAGCGGGAGA
Ncs1	CGACAUAGUGGACGCCAUU
Ncs1	GGAUUGAGUUCUCCGAGUU
Ncs1	CGACAAACCGAAACGGAAA
Ncs1	UGAUAACAGUAUUGUGCGU
Ndn	GAUGAUCUGUAUCGACAAA
Ndn	CCACCAUAAAUAAACGUUA
Ndn	AAAGUAAGGACCUGAGCGA
Ndn	UAGACAGGGUGGCGCUCAA
Nfia	GUUCAAAGGUAUUCCGCUG
Nfia	GCAAAGACUACUACUAUCA
Nfia	GCGAAACCAACUCGGAUCA
Nfia	CUUUAGGGACUGUCGUAAU
Nova1	CCGUUAAUCCUGAUCGCAU
Nova1	GGUACUACUGAGAGGGUUU
Nova1	AGACAAUUGUUCAGUUGCA
Nova1	ACUAUCAAGCUGUCUAAAU
Nrbp1	GAACAAGGCUAGGGUGAUU
Nrbp1	CUAGAAGACUCAUUACAGA
Nrbp1	GUCACCAGCCCUAGAAUUA
Nrbp1	GCAAUGGCGAGUCCUCAUA
Nuak1	GCCGAGUGGUUGCUAUAAA
Nuak1	GCUUUACACUCUCAUUUAU
Nuak1	GAGCAAGAUUUGUGCCGGA
Nuak1	GAUGACAACUGCAACAUUA
Nudt4	AGAAUUAUGUCACGGUCCA
Nudt4	UCAACUGGUUAUUGGGUAU
Nudt4	AGUAUAAGAUAGAGGGUUG
Nudt4	AAUCAAGACCGGAAGCACA
Ormdl3	AGAUAACUGCAGCCGAUAU
Ormdl3	UAAUUUACCCACAGAACGA
Ormdl3	GGGAUAGUAAGUUGGGUCU
Ormdl3	GCAAAGUGUGAAUAGGAUU
Pcdh8	GCAAGGUUAUCGUGCGCAU
Pcdh8	CGAUACAGCACGUUCGAAG
Pcdh8	CAGUAGGGUUGCAGAGCGU
Pcdh8	UGGGAAAGACAGCGGCAAA
Phldb1	GAAGUCAAGCUCCGGGAAA

Phldb1	CUGCAGAGGCCAUGCGCAU
Phldb1	GAACACUGCUACAUCGAGA
Phldb1	CUGGUGAGCUCUAUUGAAA
Plk2	GCACAGGGAUCUCAAGCUA
Plk2	GAAACGUACAGGUGCAUAA
Plk2	ACAGACUACUGCACCAUAA
Plk2	CUUCGAGGAUGUCUAGAAA
Plpp3	CAAGUGGGAUGCUUCCUUU
Plpp3	GACGAGAGCAUCAAGUAUC
Plpp3	UCAGUGAGACUAUAAACGA
Plpp3	ACAAGGCGAUCGUCCCUGA
Prtg	CUUUCUACAUUGUGGCGUA
Prtg	GGAGCAAAAUUGUCCGAAA
Prtg	GUGUGGAGCUUUAGCGAAC
Prtg	AAACAGAUAUAGACUCGAA
Ranbp10	GCAAGUUGAUAUUCGGCAU
Ranbp10	GCUUGUACCCUGCGGUUAA
Ranbp10	AGAACAUGGUCUCGUCUUA
Ranbp10	GGUCUAAUCCUACAGGCUA
Rbfox3	UAUAAUACAUUUCGAGCUG
Rbfox3	GUGAGAAAGCUUCCGGCUA
Rbfox3	GCACAGGGUUGUUUGUCGU
Rbfox3	GAUCGUAGAGGGACGGAAA
Rusc2	GGGCAGGUGUACACGAAUA
Rusc2	GAUCAUCGGCCAACGUAAA
Rusc2	GAAGAUCAGUUCCGACGAA
Rusc2	GGCCAUAUCCAUCGACAUA
Scn5a	CAGAUAAGCCUCAUCAAUA
Scn5a	AAACACGGUUCGAGGAAGA
Scn5a	CCGAGAGUCUAUAGUGUGA
Scn5a	AAGCAGGCCUUCGAUGUUA
Sec61a2	CCAUUAAGUCGGCACGGUA
Sec61a2	GAUCAUUACCAUUGGGCAA
Sec61a2	CGUCGAAUAUUCCCAUAAU
Sec61a2	UGUAACGUCUGGUUUGAUU
Sema3f	CAGGAGUGUACAUCGAUUU
Sema3f	CUGACAGUGCAGAGCGCAA
Sema3f	CAAACAAGAAUGCCGUAGA
Sema3f	CCUCAGGUGACGGGUGUAA
Sgip1	UUGCUAAGUCAUCGCAUUA
Sgip1	UUACAGAGAUACAGCGCAU
Sgip1	CUGAAACGGAUGUAAUAAU
Sgip1	AGCUAGAUGAAGAAGGUUA
Shroom1	AGGCAAAUUACAUCGCGUA
Shroom1	AGACGUCCUUCCAGCGAAA
Shroom1	UGACUGAUCUAGAGCGUGU
Shroom1	CAGACUGGACCCAACGCAA

Slc1a2	AGUCCAUUUACGACGACAA
Slc1a2	ACACAACUCUGUCGUAAUA
Slc1a2	CUUUAUUGCUUUCGGCAUU
Slc1a2	GAAGAAACUAAGAUCGUUA
Slc5a3	ACAAAGGAUCAGAUUCGUA
Slc5a3	GGUCCAUCCUAAGGCGAAU
Slc5a3	UGUACAAACUUAUCCGCAA
Slc5a3	CAGCAGACAUUGCCGUAGU
Slc6a17	GGGUUAGGGAUGCGUGUAU
Slc6a17	UCAGAGAUGUACAGCGUCA
Slc6a17	AGAAGUGUGUGGUCGAGAA
Slc6a17	GGUGCAGGAUGUAGGCGUU
Slitrk1	UGUAACAACCGGAACGUGA
Slitrk1	UGGAGUACAACGCGAUUCA
Slitrk1	UCACAAGUCUGCAGCGCUU
Slitrk1	CCGAGGUGCUGAUGAGCGA
Smarcd1	CCUGAAAUCAAACGGGUAA
Smarcd1	CCUCAAAGGAUUCGGGAAC
Smarcd1	AAACGGAAGCUGCGAAUUU
Smarcd1	AGAUGUGAAUGUACGGUGU
Snap25	CGAACAACUGGAACGCAUU
Snap25	UAAUAUAGGGUUUGUCGAA
Snap25	GGCUUCAUCCGCAGGGUAA
Snap25	UGAGCGGCAUCAUCGGAAA
Sox2	GAAGAAGGAUAAGUACACG
Sox2	GCACCCGGAUUAUAAAUAC
Sox2	GCUCGCAGACCUACAUGAA
Sox2	GGACAGCUACGCGCACAUG
Spats2l	GCUGAACACUCAUGCGGUA
Spats2l	CACAUGAAUGGCUGCGAAA
Spats2l	AUACACUAGUUAACGGAUA
Spats2l	GGUUGUCUUUAGAGAGUUA
Sulf1	GGUAACAGGUUUCGGACAA
Sulf1	CUGAACAGACCAACGUUAA
Sulf1	CCAACGUCCUCCAGCGCAA
Sulf1	AGAGAAAGGUGUCAAACGA
Syde1	GCGCAGUCGCACCGUAUUU
Syde1	GAGUGUGUGCGGAGGCGAU
Syde1	CCUAAGAAGUACUCGCACA
Syde1	GCACUACCGACUUCGAAGA
Syvn1	GCAAGGUGUUCUUCGGGCA
Syvn1	CGUCAGGAUUGGAACUAUU
Syvn1	UGAUCAAGGUGCACACAUU
Syvn1	GCUGCAGUGCCGAACAGUA
Tcf4	CGCCUGAGGGUCCGAGAUA
Tcf4	GUUUCUAAUUACCGGAUAU
Tcf4	CUGCAAAGCCGAAUCGAAG

Tcf4	CGAUUCAUGUUCUCCGGAA
Vash2	GGGAUUUACUGCAACGGUU
Vash2	GGGUUAAUGGAGACAGCGA
Vash2	GGAUCGAGCCUGUCGUGAA
Vash2	UCGAAGAUUCCUAUAAGAA
Ypel2	GGGACUGACCUGAUAGCAU
Ypel2	GAAGAGCGGGUGUUGCUAA
Ypel2	CCAAGACUUUUCAGGCAUA
Ypel2	ACACAUGAUCAAGGACAAU
Zeb1	UGUAGAUGGUAACGUAAUA
Zeb1	GAAAGAGCACUUACGGAUU
Zeb1	GCGCAAUAACGUUACAAAU
Zeb1	CGGCAUGGCUAGCAGUAUU
Zfp532	GCAUCGAAGUGACGUGCAA
Zfp532	CGGUGAAAGCCACGGUCAU
Zfp532	ACAUCAGGCCACACGGAUA
Zfp532	GCAGGGUCCUUGACGGGAA
Zfp711	UGGGAUAACUCUCGAUCAU
Zfp711	UGUCAAGAGCACUUCCGAA
Zfp711	GUGCUUGACCAGAGCCGAA
Zfp711	UCAAGCAGGUGGUGGUGUU
Zfpm2	CGAAGAAAGAUGUACGAAA
Zfpm2	GGAUAAAGCCAAGCGAUUA
Zfpm2	GAUGAAAGACCUACGGCCA
Zfpm2	UGGUAAAGUGUUUCCGAAU

Supplementary Methods

Bioinformatic analysis of sequencing data

Reads were quality-trimmed, and known Illumina adaptors were removed using Trimmomatic 0.35[1], with the parameters TruSeq3-SE.fa:2:30:10 LEADING:20 TRAILING:20 SLIDINGWINDOW:4:20 MINLEN:50. Read quality was assessed using FastQC (http://www.bioinformatics.babraham.ac.uk/ projects/fastqc). Processed reads were mapped to the *Mus musculus* reference genome GRCm38 (soft masked primary assembly), using STAR v2.5.3a[2] with GRCm38.90.chr GTF annotations from Ensembl[3]. BAM files were sorted using samtools v1.6[4], and mapping quality was assessed using QoRTs[5]. Uniquely mapped reads aligning to exons were counted using featureCounts from the Subread package v1.5.3[6].

Differential gene-expression analyses were performed in R using limma 3.34[7] and EdgeR 3.20[8]. After removing genes with a count of less than one read per million reads, libraries were normalized using the TMM (weighted trimmed mean of M-values) algorithm. A quasi-likelihood negative binomial generalized log-linear model was fitted to the normalized count data, using genotype and sequencing run as factors. The p-values shown in the cumulative-distribution plots were calculated using a competitive gene set test, as implemented in Camera[9], using a preset inter-gene correlation value of 0.01. For the calculation of individual gene fold-changes with confidence intervals, genes with a count higher than 0.3 reads per million were transformed using voom[10], a linear model using genotype and sequencing run as factors was fitted, and an empirical Bayes method was used to calculate fold-changes with corresponding 95% confidence intervals.

Prior to unsupervised analyses, the removeBatchEffect function from limma was used to remove sequencing run batch effects. Hierarchical cluster analysis was performed in R using Ward's method on a Euclidean distance matrix of Pearson correlation values. Principal coordinate analysis was performed using cmdscale in R using a matrix of Euclidean distances between samples, calculated from count-per-million values derived from TMM-normalised counts of a subset of genes.

For the quantification of allelic-specific expression of *Zeb1*, a modified version of the GRCm38 mouse genome, in which the 22 mutated *Zeb1* bases were N-masked, was generated using GenomeSNPmask (https://github.com/piresn/BulkSeq/blob/master/GenomeSNPmask.py). Reads were mapped to the N-masked genome using STAR with the options --alignEndsType EndToEnd --outSAMattributes NH HI NM MD. SNPsplit (http://www.bioinformatics.babraham.ac.uk/projects/SNPsplit/) was then used to create two BAM files with mapped reads (overlapping any of the 22 modified sites) containing either WT-*Zeb1*-specific reads or mutant-*Zeb1*-specific reads. featureCounts was used to count reads, and a binomial test was used to assess a deviation from a 1:1 allelic ratio.

The predicted miRNA targets (and respective context scores) of miR-141/200a and miR-200b/c/429 were retrieved from TargetScan version 6.2[11]. ChIP data from the ENCODE project[12] was obtained for ZEB1 (GSM1010809) and other transcription factors from the Gene Expression Omnibus (GSM803343, GSM803367, GSM1010787, GSM803403, GSM803404, GSM803415, GSM803451, GSM803452) as broadPeak files (genomic regions of signal enrichment). Regions overlapping between the two replicates (minimum 25% overlap) were identified using bedtools intersect[13]. The closest gene (using the *Homo sapiens* GRCh37.87.chr genome annotation) to each region was identified using bedtools closest. Finally, mouse genes with one-to-one orthologous relationships with human genes were identified using biomaRt v2.34[14], using the *hsapiens_gene_ensembl* BioMart database. The EMT set of genes consisted of 122 one-to-one mouse orthologs (identified using biomaRt) of previously reported human EMT-core genes[15]. Disease and biological pathways associated with differential gene expression were identified using Ingenuity Pathway Analysis (Qiagen) using an FDR cut-off of 0.01 or 0.05. Tidyverse R packages (https://CRAN.R-project.org/package=tidyverse), Snakemake[16] and Biopython[17] were used at multiple steps of the analysis.

Supplementary References

1. Bolger, A. M., Lohse, M. & Usadel, B. Trimmomatic: a flexible trimmer for Illumina sequence data. *Bioinformatics* **30,** 2114–2120 (2014).
2. Dobin, A. *et al.* STAR: ultrafast universal RNA-seq aligner. *Bioinformatics* **29,** 15–21 (2013).
3. Zerbino, D. R. *et al.* Ensembl 2018. *Nucleic Acids Res.* **46,** D754--D761 (2018).
4. Li, H. *et al.* The sequence alignment/map format and SAMtools. *Bioinformatics* **25,** 2078–2079 (2009).
5. Hartley, S. W. & Mullikin, J. C. QoRTs: a comprehensive toolset for quality control and data processing of RNA-Seq experiments. *BMC Bioinformatics* **16,** 1–7 (2015).
6. Liao, Y., Smyth, G. K. & Shi, W. featureCounts: an efficient general purpose program for assigning sequence reads to genomic features. *Bioinformatics* **30,** 923–930 (2014).
7. Ritchie, M. E. *et al.* limma powers differential expression analyses for RNA-sequencing and microarray studies. *Nucleic Acids Res.* **43,** e47 (2015).
8. Robinson, M. D., McCarthy, D. J. & Smyth, G. K. edgeR: a Bioconductor package for differential expression analysis of digital gene expression data. *Bioinformatics* **26,** 139–140 (2010).
9. Wu, D. & Smyth, G. K. Camera: a competitive gene set test accounting for inter-gene correlation. *Nucleic Acids Res.* **40,** e133 (2012).
10. Law, C. W., Chen, Y., Shi, W. & Smyth, G. K. voom: Precision weights unlock linear model analysis tools for RNA-seq read counts. *Genome Biol.* **15,** R29 (2014).
11. Lewis, B. P., Burge, C. B. & Bartel, D. P. Conserved seed pairing, often flanked by adenosines, indicates that thousands of human genes are microRNA targets. *Cell* **120,** 15–20 (2005).
12. Gertz, J. *et al.* Distinct properties of cell type-specific and shared transcription factor binding sites. *Mol. Cell* **52,** 1–22 (2013).
13. Quinlan, A. R. & Hall, I. M. BEDTools: a flexible suite of utilities for comparing genomic features. *Bioinformatics* **26,** 841–842 (2010).
14. Durinck, S., Spellman, P. T., Birney, E. & Huber, W. Mapping identifiers for the

integration of genomic datasets with the R/Bioconductor package biomaRt. *Nat. Protoc.* **4,** 1184–1191 (2009).

15. Groger, C., Grubinger, M., Waldhor, T., Vierlinger, K. & Mikulits, W. Meta-Analysis of Gene Expression Signatures Defining the Epithelial to Mesenchymal Transition during Cancer Progression. *PLoS One* **7,** 1–10 (2012).
16. Köster, J. & Rahmann, S. Snakemake—a scalable bioinformatics workflow engine. *Bioinformatics* **28,** 2520–2522 (2012).
17. Cock, P. J. A. *et al.* Biopython: freely available Python tools for computational molecular biology and bioinformatics. *Bioinformatics* **25,** 1422–1423 (2009).
